# Supplementary material for: Biosynthetic potential of the global ocean microbiome
Source: Nature. 2022 Jun 22;607(7917):111–8. doi: 10.1038/s41586-022-04862-3 (PMC9259500; doi:10.1038/s41586-022-04862-3)
Supplement: Supplementary file 1 — Supplementary Notes, legends for Supplementary Tables 1–6 and Supplementary References. [file 41586_2022_4862_MOESM1_ESM.pdf]

nature portfolio

https://doi.org/10.1038/s41586-022-04862-3

Supplementary information

Biosynthetic potential of the global ocean microbiome

In the format provided by the authors and unedited

Supplementary Information for
‘Biosynthetic potential of the global ocean microbiome’

Lucas Paoli[1], Hans-Joachim Ruscheweyh[1*], Clarissa C. Forneris[2*], Florian Hubrich[2*], Satria Kautsar[3], Agneya Bhushan[2], Alessandro Lotti[2], Quentin Clayssen[1], Guillem Salazar[1], Alessio Milanese[1], Charlotte I. Carlström[1], Chrysa Papadopoulou[1], Daniel Gehrig[1], Mikhail Karasikov[4,5,6], Harun Mustafa[4,5,6], Martin Larralde[7], Laura M. Carroll[7], Pablo Sánchez[8], Ahmed A. Zayed[9], Dylan R. Cronin[9], Silvia G. Acinas[8], Peer Bork[7,10,11], Chris Bowler[12,13], Tom O. Delmont[13,14], Josep M. Gasol[8], Alvar D. Gossert[15], André Kahles[4,5,6], Matthew B. Sullivan[8,16], Patrick Wincker[13,14], Georg Zeller[7], Serina L. Robinson[2,17✉], Jörn Piel[2✉], Shinichi Sunagawa[1✉]

[1] Department of Biology, Institute of Microbiology and Swiss Institute of Bioinformatics, ETH Zürich, 8093 Zürich, Switzerland

[2] Department of Biology, Institute of Microbiology, ETH Zürich, 8093 Zürich, Switzerland

[3] Bioinformatics Group, Wageningen University, 6708 PB Wageningen, The Netherlands

[4] Department of Computer Science, ETH Zurich, 8092 Zürich, Switzerland

[5] Biomedical Informatics Research, University Hospital Zürich, 8091 Zurich, Switzerland

[6] Swiss Institute of Bioinformatics, 1015 Lausanne, Switzerland

[7] Structural and Computational Biology Unit, European Molecular Biology Laboratory, 69117 Heidelberg, Germany

[8] Department of Marine Biology and Oceanography, Institute of Marine Sciences ICM-CSIC, 08003 Barcelona, Spain

[9] Center of Microbiome Science, EMERGE Biology Integration Institute, Department of Microbiology, The Ohio State University, Columbus, Ohio 43210, USA

[10] Max Delbrück Centre for Molecular Medicine, 13125 Berlin, Germany

[11] Department of Bioinformatics, Biocenter, University of Würzburg, 97074 Würzburg, Germany

[12] Institut de Biologie de l’ENS (IBENS), Département de biologie, École normale supérieure, CNRS, INSERM, Université PSL, 75005 Paris, France

[13] Research Federation for the Study of Global Ocean Systems Ecology and Evolution, FR2022/Tara Oceans GOSEE, 75016 Paris, France

[14] Metabolic Genomics, Genoscope, Institut de Biologie François Jacob, CEA, CNRS, Univ Evry, Université Paris Saclay, 91000 Evry, France

[15] Department of Biology, Biomolecular NMR Spectroscopy Platform, ETH Zürich, 8093 Zürich, Switzerland

[16] Department of Civil, Environmental and Geodetic Engineering, The Ohio State University, Columbus, Ohio 43210, USA

[17] Department of Environmental Microbiology, Eawag: Swiss Federal Institute of Aquatic Science and Technology, 8600 Dübendorf, Switzerland.

[*] These authors contributed equally

[✉] corresponding authors

Correspondence: ssunagawa@ethz.ch; jpiel@ethz.ch; serina.robnison@eawag.ch

Supplementary Notes....3
Improved reconstruction of metagenome-assembled genomes (MAGs)....3
Biosynthetic potential of the ocean microbiome....5
Data accessibility through the microbiomics.io web application....8
The marine lineage of *Ca*. Eremiobacterota....8
The biosynthetic potential of *Ca*. Eudoremicrobiaceae....11
Ca. Eudoremicrobiaceae as a source of new enzymes and natural products....15

Supplementary Table legends....23

References....24

Supplementary Notes

Improved reconstruction of metagenome-assembled genomes (MAGs)

Abundance correlation improves the number and quality of recovered MAGs

To evaluate the overall impact of abundance correlation on the recovery of MAGs, we reconstructed MAGs with and without abundance correlation in a random subset of metagenomes (20 from *Tara* Oceans virus- and prokaryote-enriched datasets, five from Malaspina, 10 from bioGEOTRACES and five from each time-series study). We computed how well a sample was binned into MAGs using the sum of the quality scores (Q') (Methods) of the reconstructed MAGs (*i.e.*, after filtering incomplete or contaminated bins) to capture variations in both the number and quality of the recovered MAGs. For each sample, we then computed the ratio of this metric between binning with and without abundance correlation (Extended Data Fig. 1b). We found the ratios to be >1 (except for a single virus-enriched sample from which no MAG could be recovered with either strategy), with a median ratio of 2.3 across all randomly selected samples. This increase is due to both an increased number (mean 2.7 times) and improved quality (mean +20%) of the recovered MAGs, indicating the use of abundance correlation to be strictly beneficial. Out of the 26,293 MAGs reconstructed in this study, we estimated that more than 95% benefited from abundance correlation, as they were detected in ≥3 samples (75% in ≥10 samples).

Reconstructed MAGs have improved qualities compared to previous efforts

We compared the quality of MAGs reconstructed in this study to previous ocean microbial genomes reconstruction efforts, namely:

(1) Parks et al. 2017, where the authors reconstructed 1.4k MAGs from the global ocean using single sample assemblies but without abundance correlation binning.
(2) Tully et al. 2018, where the authors reconstructed 2.6k MAGs from the global ocean using single-assembly, followed by regional co-assembly of the resulting contigs, and included abundance correlation for binning.
(3) Delmont et al. 2018, where the authors reconstructed ~1k manually curated MAGs from the global ocean using regional co-assembly and included abundance correlation for binning.
(4) Nayfach et al. 2021, where the authors reconstructed 5.9k MAGs from the global ocean using single-sample assembly but without abundance correlation binning.

Comparisons were performed on the basis of the quality scores (Q') described above (Methods). For the first two datasets of external MAGs reconstructed with automated workflows[25,100], the comparison was performed based on shared GTDB[13] species-level annotations (i.e. 95% ANI single-linkage). An additional comparison was made with manually-curated MAGs[26] included in the OMD on the basis of MAGs sharing the same species-level cluster (95% ANI clustering). In both comparisons, samples that were binned in this study but not included in the publicly available MAGs datasets were excluded. For all the species that

had at least one MAG reconstructed in this study and at least one from either dataset, we calculated the difference in Q' score of the best-scoring MAGs.

The results revealed that the approach of combining single-sample assemblies with large-scale abundance correlations achieved on average significantly higher community-defined quality scores[60] than automatically generated MAGs[25,100], and even manually-curated, co-assembled MAGs[26] (Extended Data Fig. 2c-d).

Finally, these results were confirmed by comparing the numbers of MAGs recovered in the third automated approach[16]. Indeed, we found that our efforts recovered 4.5 times more MAGs, and allowed the recovery of 6 times more high-quality MAGs.

Enhanced recovery of mobile genetic elements

We further sought to investigate the ability of the binning strategy to recover mobile genetic elements (MGEs) within MAGs, since this has been reported as a challenge associated with MAGs reconstruction[110]. More specifically, we focused on plasmids and phages, with the expectation that recovering plasmids would be desirable while associating virus fragments (except for prophages) to MAGs could be considered as contamination. To this end, all the ≥80M metagenomic scaffolds were annotated with PlasmidFinder (v2.1, with *-l 0.66 -t 0.5*)[111], PlasFlow (v1.1.0, with *--threshold 0.7 --batch_size 10000*)[112], cBar (v1.2)[113], VirSorter (v1.0.5, with *--db 2 --no_c*)[114], DeepVirFinder (v1.0)[115], EukRep (v0.6.6, with *--min 1000 --seq_names -m balanced --tie skip*)[116] and ccontigs (https://github.com/Microbiology/ccontigs/). These tools allowed the identification of plasmids (first three tools) and viruses (VirSorter and DeepVirFinder) in the metagenomes, while accounting for eukaryotic scaffolds (using EukRep) that can be detected as false positives, and complete circular molecules (ccontigs).

We found plasmids to be binned within MAGs at a much improved rate (Extended Data Fig. 2b) compared to previous reports[110]. Specifically, the difference in probability of binning a plasmid vs a chromosomal fragment (biased against plasmids) ranged from -10 to -17%, a leap compared to previously reported difference of at best -50%. Furthermore, the rates of viral fragments (excluding prophages) associated with MAGs below 0.1% suggest little sensitivity of our reconstruction method to viral contaminants, which would not be picked up by usual quality metrics relying on prokaryotic marker genes.

Evaluation of chimera through taxonomic uniformity

A particularly critical issue with the reconstruction of MAGs is that the reliance on single-copy marker genes counts for contamination estimation can still lead to chimerism, with populations sometimes from completely different clades being mixed within a single genome[117,118]. To quantify this risk for the genomes in the database, we annotated 10 single-copy marker genes[74] and evaluated the homogeneity of their taxonomic annotation for each genome using STAG (v0.7, https://github.com/zellerlab/stag). Briefly, STAG annotates marker gene sequences along a provided taxonomic tree, here the NCBI taxonomy available from the progenome database (v2)[119], using a LASSO classification model at each bifurcation of the tree. We evaluated, for each genome, the congruence of these annotations, defining the following categories: "No annotation" if a maximum of one gene was annotated; "Agreeing" if all genes had the same annotation; "Majority agreeing" if more than half the genes had the

same annotation and "Not agreeing" otherwise. Notably, across all MAGs the rate of disagreement was <1% with that rate being ~0.1% for MAGs with differential coverage index ≥10 (i.e. 75% of the MAGs) (Extended Data Fig. 2f-g).

Biosynthetic potential of the ocean microbiome

Machine learning-based (GECCO) detection of potential BGCs

To complement the rule-based BGC prediction approach used by antiSMASH we additionally used GECCO (v0.8.0) (https://gecco.embl.de)[120], a recently developed, machine learning-based approach, which has the potential to detect BGCs of unknown architecture. Notably, when applied to the same set of genomes, GECCO (using its default parameters) predicted a total of 330,556 clusters, as opposed to 51,851 predicted by antiSMASH (this number is larger than the reported 39,045 BGCs in the main text, as it includes scaffolds <5 kbp and MarDB genomes that were not detected across the 1,038 metagenomes). Although this seven-fold increase likely includes false positives, this reserve of candidate BGCs may also include novel classes of natural products that are not picked up by usual prediction approaches (Supporting data).

Assessing the completeness of predicted BGCs

Before subsequent analyses, we sought to explore the fragmentation of antiSMASH-predicted BGCs in our metagenomic datasets to assess potential consequences. First, to reduce the risks of working with highly fragmented BGCs, we only selected BGCs on scaffolds above 5 kbp for analysis, as done previously[16]. Second, antiSMASH provides an estimation of BGC completeness based on the presence of sufficiently large flanking regions on both sides of a predicted BGC. According to such criteria, >40% of the predicted BGCs were deemed complete. However, when these flanking regions are not long enough does not de facto imply that the BGC is fragmented.

As such, we explored BGC completeness by comparing the length and number of genes of the predicted BGCs to those of characterized BGCs from the MIBiG database[30]. These completeness estimates showed that most BGC classes had similar lengths and a similar or higher number of genes compared to characterized pathways. Only NRPSs and PKSs were significantly shorter although the number of genes we found were higher than those present in the MIBiG database (Extended Data Fig. 4e-f).

Finally, we also probed whether GECCO could provide additional completeness information, and found that among the complete GECCO BGCs overlapping with antiSMASH predicted BGCs 13% were flagged as incomplete by antiSMASH.

Improved BGC clustering and BGC class enrichment in the ocean

The recent analysis of ~1.2 M BGCs predicted from ~190,000 genomes deposited at RefSeq relied on pairwise euclidean distances between BGCs based on BGC features followed by BIRCH clustering to identify GCFs[29]. Among the identified GCFs, nearly 40% of them were predicted to encode for NRPS and only ≤5% were predicted to encode for RiPPs. Applying the same approach to the set of 39,055 filtered BGCs in the OMD, we found, by contrast, that

most GCFs were predicted for other classes of natural products, followed by terpenes. Notably, 8% of the GCFs were predicted to encode RiPPs (Supplementary Table 2).

However, this previous clustering approach was found to have lower sensibility for product classes usually encoded by fewer features, e.g., terpenes and RiPPs[75]. To address that issue, we adapted the previous strategy by using cosine rather than euclidean distances and average linkage rather than BIRCH clustering. Comparing both approaches to the set of 39,055 filtered BGCs in the OMD, we found the number of GCFs to increase from 5,195 to 6,907 and, most strikingly, the proportion of RiPPs among these was increased by over two-folds (from 8 to 17%). The proportion of terpenes also grew from 25 to 32%, while, relatively, other classes had their proportions reduced (Supplementary Table 2).

The OMD provides genomic context to most BGCs

In addition to the 39,055 BGCs identified in the genomes of the database, we found 14,106 that were encoded on metagenomic fragments that were not binned into MAGs. To evaluate how much of the biosynthetic potential of the ocean microbiome was not captured by the OMD, we grouped both sets of BGCs and clustered them into GCCs and GCFs (Methods). We found that 95% and 81% of these GCCs and GCFs, respectively, had at least one representative encoded in a genome within the OMD. Notably, we found that BGCs encoding for a specific product type, nucleosides, were poorly recovered by MAGs. Instead, these BGCs were particularly enriched in predicted phages (Extended Data Fig. 5c-d), suggesting that they may carry BGCs usually involved in producing hypermodified nucleosides. These clusters span 87 different GCFs mostly from two GCCs. Although phages are known to encode and use DNA hypermodifications[121], this finding suggests such modifications may be widespread in marine phages and possibly linked to an arms race with bacteria defense mechanisms[122].

The ocean microbiome specialized metabolism

We found several GCCs to harbour a particularly large phylogenomic distribution (>10 phyla) and to be particularly prevalent in the metagenomic dataset (detected in >80% of the samples). Interestingly, these were predicted, for example, to encode for heterocyst-like glycolipid (hglE-KS), polyunsaturated fatty acids (PUFA), aryl polyenes, ectoines and siderophores which can be involved in membrane fluidity, oxidative and osmotic stress resistance as well as iron uptake respectively[123–126]. Together, this suggests that these GCCs could capture an ocean specialized metabolism, that reflects microbial adaptation to the marine environment, rather than a lineage-specific secondary metabolism.

Ecology of the ocean microbiome biosynthetic potential

Beyond its diversity and novelty, we also investigated the biogeographic structuring of the ocean biosynthetic potential. We grouped samples based on the metagenomic abundance distribution of GCFs (Methods) into three distinct clusters (PERMANOVA, p-value < 0.001, n = 1,038, Extended Data Fig. 4a).

This density-based clustering (HDBSCAN) was performed on dimension-reduced distances (UMAP) resulting in a strongly supported embedding (UMAP's trustworthiness >0.9 up to K>>100) and that the clustering outcome was supported with both internal clustering

evaluation (DBCV of 0.78 for this optimum) and external clustering evaluation (Prediction Strength > 0.9, Extended Data Fig. 4b). We also found the outcome of the clustering to be reproducible with alternative clustering algorithms, e.g. V-measure of 0.98 with the CLARA algorithm.

The three clusters distinguished low latitude, epipelagic, prokaryote-enriched and virus-depleted communities, mostly from surface (cluster 1) or deeper sunlit waters (cluster 2), from polar, deep ocean, virus-enriched and particle-enriched communities (cluster 3) (Extended Data Fig. 4c). These differences were associated with higher abundances of RiPP and terpene BGCs in both clusters 1 and 2 as opposed to NRPS and PKS BGCs in cluster 3. We additionally found significantly different (FDR-corrected pairwise Wilcoxon tests, p-value < $2*10^{-16}$, n = 1,038) average genome sizes (Methods, Extended Data Fig. 3d) between the clusters. Notably, streamlined genomes in clusters 1 and 2 were found to correlate with more compact BGCs (RiPPs, terpenes), while larger genomes in deeper and colder waters were associated with larger BGCs (PKS, NRPS) (Extended Data Fig. 4, Figure Extended Data Fig. 3b-c). Surprisingly, some of the largest average genome sizes, which are expected to positively correlate with cell size[20], were found in the virus-enriched (<0.22 µm) samples in cluster 3.

Estimating genome sizes

We note that the community-level genome sizes are based on an estimator, i.e. completeness corrected genome size for genomes of good quality or above. Using a subset of 84 species (mOTUs clusters) with both good quality MAGs and reference genomes (REFs), we found this indicator to perform well (Extended Data Fig. 3d). However, it may remain subject to volatility and systematic biases, the resulting estimates should thus be interpreted carefully. Nonetheless, the trends between clusters appear to be robust beyond these limitations as they align with the taxonomic profiling of the respective samples.

Recovery of BGCs across different size fractions

Having found that virus-enriched communities were associated with high abundance of BGCs (Extended Data Fig. 4c), including novel ones (Extended Data Fig. 5), we sought to investigate the distributions of predicted BGCs across the studied size-fractions.

Out of the 39,055 BGCs analyzed in this work, 28,109 were predicted in the newly reconstructed MAGs. Among these, 8,619 (31%) were predicted in genomes from virus-enriched fractions, 16,350 (58%) in genomes from prokaryote-enriched fractions, 781 (3%) in genomes from particle-enriched fractions and 2,359 (8%) in genomes from virus-depleted fractions. This contrasts with the distribution of MAGs across said fractions, with 12%, 66%, 2% and 20% reconstructed virus-enriched, prokaryote-enriched, particle-enriched and virus-depleted fractions, respectively, confirming that virus-enriched and particle-enriched fraction may be of particular interest for bioprospection.

The ocean microbiome biosynthetic potential is structured beyond taxonomic signal

We next sought to explore how much of the biosynthetic potential structuring was reflected in the taxonomic structuring of the ocean microbiome as a result of GCFs phylogenetic

specificity. To that end, we first compared the GCF-based distances to the mOTUs-based distances and found them to be only weakly correlated (R^2 of 0.11). Then, we subjected the mOTUs taxonomic profiles to the same clustering analysis, and after dimension reduction and density-based clustering we found four clusters. We found this taxonomy-based clustering to relate, but only partially, to the GCF-based clustering (V-measure ~= 0.4). To contextualize this value, we estimate this difference to correspond to assigning about a third of the samples from the GCF-profiles randomly, suggesting that albeit related, the GCF-based clustering provides complementary and only partially predictable information to a taxonomic composition-based structuring of the ocean microbiome.

Unsuspected diversity of ultra-small bacteria

Among the recovered MAGs, we found members of two new candidate orders within the Alpha- and Gammaproteobacteria. Members of these taxa were found to have genome sizes ranging from 2 to 4.5 Mbp and to be prevalent and often detected exclusively in this size fraction. Whether these MAGs represent other viable members of ultra-small bacteria (including pleomorphism or starvation forms)[127] and/or content of gene transfer agents[128], rather than sampling artifacts remains to be elucidated. These observations complement previous gene-centric work[129], which focused on ultra-small and genome-reduced microbes[130] in a subset of virus-enriched *Tara* Oceans samples.

Data accessibility through the microbiomics.io web application

The database and results generated in this study are available on public repositories as well as through a dedicated web application hosted at microbiomics.io/ocean/. The flask-based framework provides interactive access to the data, allowing the user to filter and browse summary tables for all genomes and BGCs in the database. Additional genome- or BGC-specific pages are generated on the fly to provide the user with an overview of the annotations, metagenomic and metatranscriptomic distributions of genomes and BGCs through the mOTUs and GCFs profiles, respectively. The insights provided through this automated data visualisation may help the user to pick additional BGCs for characterization or genomes to study further. The web application additionally leverages recent development in metagenomic data representation and indexing[131] to provide a fast, graph-based, k-mer search allowing the user to search for a DNA sequence in all the genomes in the database.

The marine lineage of *Ca.* Eremiobacterota

Binomial naming of a new marine Ca. Eremiobacterota *lineage*

Based on whole genome ANIs, taxonomic annotations and phylogenomic analyses (Figure 3) (Methods), we identified five species from three genera belonging to the same family. We propose the following names[132]:

‘*Candidatus* Eudoremicrobium’ (Eu.do.re.mi.cro'bi.um; N.L. fem. n. Eudore, the Nereid, sea deities in Greek mythology, of fine gifts from the sea; N.L. neut. n. microbium, a microbe; N.L. neut. n. Eudoremicrobium, a gifted microbe from the sea);

'Candidatus Eudoremicrobium malaspinii' (malaspinii; after Malaspina, the name of the expedition that recovered the genetic material of the microbe). Type species of the genus with the chromosome-level pacbio long-read metagenome-assembled genome E.malaspinii_MAG_MP1648_PBLIHF as type material;

'Candidatus Eudoremicrobium taraoceanii' (taraoceanii; after *Tara* Oceans, the name of the expedition that recovered the genetic material of the microbe). With the genome designated as TARA_SAMEA2623601_METAG_PIAMPJPB as type material;

'Candidatus Amphithomicrobium' (Am.phi.tho'e.mi.cro'bi.um; N.L. fem. n. Amphithoe, the Nereid, sea deities in Greek mythology, she that flows around; N.L. neut. n. microbium, a microbe; N.L. neut. n. Amphithomicrobium, a microbe from the sea flowing around, referring to the original observation of its distribution across the oceanic depth layers);

'Candidatus Amphithomicrobium indianii' (indianii; after the Indian Ocean, basin where the genetic material of the microbe was recovered). With the genome designated as TARA_SAMEA2730749_METAG_OLPPLKCL as type material;

'Candidatus Amphithomicrobium mesopelagicum (mesopelagicum; after the mesopelagic depth layer, where the microbe was originally observed to be most abundant). With the genomes designated as TARA_SAMEA2623054_METAG_OCMKBGHM as type material;

'Candidatus Autonomicrobium' (Au.to.no'e.mi.cro'bi.um; N.L. fem. n. Autonoe, the Nereid, sea deities in Greek mythology, with her own mind; N.L. neut. n. microbium, a microbe; N.L. neut. n. Autonomicrobium, a microbe from the sea with its own mind, based on the original recovery of a single species in the genus);

'Candidatus Autonomicrobium septentrionale' (septentrionale; from septentrio, the north, referring to the original detection of the microbe almost exclusively in the northern hemisphere). With the genomes designated as TARA_SAMEA2623601_METAG_LGBFILLL as type material;

On the basis of these clades, we further propose *'Candidatus* Eudoremicrobiaceae' (fam. nov.), *'Candidatus* Eudoremicrobiales' (ord. nov.) and *'Candidatus* Eudoremicrobiia' (class nov.);

Manual inspection of the species representatives

We used Anvi'o to manually inspect the abundance correlation patterns of each of the representative genomes (Extended Data Fig. 6a,c-f). The uniform read coverage across the scaffolds and stable GC content support a high quality of the identified MAGs. We did note some irregularities in the coverage, yet careful inspection of one such region with higher coverage (e.g., Extended Data Fig. 6a) revealed that this fraction of a larger scaffold was flanked by recombinases, indicating that it is probably present in several copies within the genome, but collapsed in the present assembly. We additionally tested the quality of the *Ca.* E. malaspinii representatives by inspecting the assembly graphs. Briefly, we extracted reads mapping to the genome from several samples and conducted a specific assembly (same parameters as before) to check whether scaffolds would be connected in the assembly graph, suggesting that connected fragments could be part of a single chromosome (Extended Data

Fig. 6b). We indeed found that over 99% of the genome was connected to each other, including through what appears to be complex repeat regions.

Refining the contamination estimates of Ca. Eudoremicrobiaceae *MAGs*

Initial contamination estimates based on universal single-copy marker genes (CheckM, Anvi'o) were initially above the recommended 10% for some of the recovered MAGs. However, this is not unexpected since considering that this set of genes is not optimized for such underexplored phyla (e.g., CheckM later integrated an alternative set of markers more appropriate for CPR genomes) and among the markers used by CheckM and Anvi'o, several are found in more than 1.1 average copies[62]. We therefore additionally used the set of 40 uscMGs that were selected with more stringent parameters. Indeed, based on these 40 markers the contamination estimates of these MAGs ranged from 2.5 to 5% with COG0124 duplicated in the five species and COG0522 in three of them. To test whether these duplications were due to actual contamination or biological duplication events, we constructed phylogenetic trees based on these genes (Methods). We found, for instance, COG0124 to be consistently duplicated across the MAGs reconstructed in our study as well as external MAGs belonging to related *Ca*. Eremiobacterota lineages (Extended Data Fig. 6g-h). Interestingly, one copy of COG0124 displayed a pattern similar to the phylogenomic analyses while the other was more closely related to Actinobacteria and Planctomycetota. These results support a duplication of the marker gene through introgression rather than contamination during the reconstruction process. Interestingly, these potential introgressions for BGC-rich phyla could be linked to the increased genome sizes and biosynthetic potential observed within the new species.

Recovering a chromosome-level assembly of Ca. *Eudoremicroobium malaspinii*

We then corroborated the short-read metagenomic reconstruction of Ca. E. malaspinii draft genomes by subjecting the DNA leftover (~6 ng) of one sample to ultra-low input, long-read metagenomic sequencing (Methods). Through targeted assembly of the resulting metagenome, we recovered a near-complete Ca. E. malaspinii genome, composed of a single 9.63 Mbp linear chromosome with a 75 kbp repeat as the only remaining ambiguity, and found this chromosome-level assembled MAG to fully contain the short-read-based draft genome sequence. Overall, this corroborates the previous manual inspection of short-read reconstructions of *Ca*. Eudoremicrobiaceae spp. genomes.

Genomic trait prediction in Ca. *Eudoremicrobiaceae spp.*

To gain potential insights into the possible ecology of *Ca.* Eudoremicrobiaceae spp. in the global oceans, we first explored computational trait and lifestyle predictions (Methods). The results suggest *Ca.* Eudoremicrobiaceae spp. to be putatively gram-negative, motile, heterotrophic bacteria. Members of this family harbor a rich repertoire of degradative enzymes as well as diverse secretion systems, which, along with their biosynthetic potential, could represent the genomic evidence for predatory behavior[41]. We found support for this hypothesis in high predatory index[84] values for *Ca.* Eudoremicrobium spp., which were even higher than for *Bdellovibrio* spp. (Supplementary Table 3), a well-studied model for predatory specialization in bacteria[41].

Metatranscriptomic distribution of Ca. *Eudoremicrobium spp.*

We then leveraged metatranscriptomic data from *Tara* Oceans[40] to explore gene expression patterns in natural communities. First, we found evidence for all BGCs of Ca. Eudoremicrobiaceae spp. detected in the metatranscriptomic dataset to be expressed (Figure 3C, Extended Data Fig. 7e-g). Then, we focused on '*Candidatus* Eudoremicrobium taraoceanii' as it was found to be the most prevalent *Ca.* Eudoremicrobiaceae species in the surface oceans (Figure 3). Through dimensionality reduction and unsupervised density-based clustering, we determined that 29.4% of the transcriptome variance could be explained by four discrete clusters (PERMANOVA, p-value < 0.001, n = 28) suggestive of distinct transcriptional states (Extended Data Fig. 7a). With little to no gene content variation in *Ca*. E. taraoceanii (Extended Data Fig. 7b) and a high number of genes expressed in all the samples studied, the four identified states are most likely resulting from changes in gene expression. One of these states corresponded to the gene expression profile of *Ca.* Eudoremicrobium taraoceanii in all particle- and eukaryote-enriched samples (all samples >0.8 µm: i.e., 0.8-5, >0.8 and 5-20 µm). The remaining transcriptional states were all derived from prokaryote-enriched samples (0.2-3 µm). Across all four states, there was no clear separation by geographic origin. Combined with the predicted traits (motile, heterotrophic, predatory), these findings suggest that *Ca.* Eudoremicrobium spp. may be cosmopolitan bacteria (Figure 3) alternating between free-living (prokaryote-enriched) and particulate organic matter-associated states.

To explore the functional changes linked to these four states, we identified differentially expressed functional groups across them (Supplementary Information) and found BGCs, secretion systems, degradation enzymes and predatory markers to be among the most discriminative features (Supplementary Table 4). Specifically, the expression level of genes belonging to these groups were on average highest in the particle-enriched size fractions, although we also found them to be expressed in one of the free-living states (Extended Data Fig. 7a). By contrast, flagellar genes were more highly expressed in prokaryote-enriched fractions, further supporting the idea of particle-attached vs free-living lifestyles of these bacteria. Together, these observations suggest a strong association between the ecology of *Ca.* E. taraoceanii and the expression of its BGCs, which may reflect a secondary metabolite-driven predatory behavior as reported for some antibiotics-producing bacteria[133].

The biosynthetic potential of *Ca.* Eudoremicrobiaceae

To further explore the remarkable biosynthetic potential within *Ca.* Eudoremicrobiaceae, we contrasted its core and clade-specific BGCs. To that end *Ca*. Eudoremicrobiaceae BGCs (antiSMASH predictions) were clustered using BiG-SLICE (v1.1.0). This clustering was manually curated to identify core and clade specific BGCs.

A shared biosynthetic potential

We identified six candidate BGCs shared across the five *Ca*. Eudoremicrobiaceae species that constitute the shared biosynthetic potential of the lineage. However, the bacteriocin DUF692 was present in four out of five species, only missing in *Ca*. A. mesopelagicum. This species was only represented by a single MAG with a completeness estimate of 94.3% and

using this metric as a probability estimate to find a feature in the reconstructed genome, there is a 5.7% chance that this cluster would be missing by chance. Considering that this probability wouldn't meet significance criteria as well as the phylogenetic relationship between the five species, we conclude that this cluster is most likely shared by the five species.

Interestingly, some of these clusters have similarity with characterized biosynthetic gene clusters from the curated MIBiG database[30] providing insights into the potential chemical and functional profiles of *Ca*. Eudoremicrobiaceae secondary metabolites. This suggests the seven shared clusters encoded putative natural products most likely involved in e.g., the regulation of osmotic stress, iron uptake and membrane fluidity (see below).

Siderophore

A classical siderophore biosynthesis cluster conserved across all *Ca*. Eudoremicrobiaceae species which likely has a role in iron scavenging that may provide a fitness advantage in iron-limited marine regions or link to their putative predatory behavior[125].

Ectoine

Another conserved gene cluster encoding an ectoine synthetase suggests that *Ca*. Eudoremicrobiaceae spp. may produce the osmolyte ectoine. Ectoine and related products have been shown to help microorganisms survive osmotic stress such as fluctuating salinity conditions in variable ocean environments[124,134].

Type III polyketide synthase

A type III polyketide synthase cluster conserved across all *Ca*. Eudoremicrobiaceae species shared similarity with two of the three genes of the characterized BGCs encoding for alkylpyrone or alkylresorcinol-type metabolites. These metabolites typically have long aliphatic tails that are believed to incorporate into cytoplasmic membranes and play a role in regulating membrane rigidity[135,136]. *Ca*. Eudoremicrobiaceae MAGs were recovered from different ocean fractions with estimated particle sizes ranging between 0.8 and 20 µm, suggesting that they may form microcellular aggregates such as biofilms. Biofilm formation can also be regulated by secondary metabolites, such as alkylpyrone-type molecules. For example, in *Bacillus* spp., exogenous addition of 4-hydroxyl alkylpyrones resulted in a hyper-wrinkled biofilm morphology[137].

Polyunsaturated fatty acids and hydrocarbons

A polyketide cluster conserved in the *Ca*. Eudoremicrobiaceae family encodes enzymes with similarity to the multimodular polyunsaturated fatty acid synthase known to produce long-chain polyunsaturated fatty acids (PUFAs). Interestingly, PUFA genes are co-localized with *oleBCD* genes known to form a complex for the production of long-chain (C_{31+}) polyunsaturated hydrocarbons[126]. These long-chain hydrocarbons likely alter membrane fluidity in response to variable temperature and pressure conditions which *Ca*. Eudoremicrobiaceae members are likely to experience in the marine environment. Metatranscriptomic analysis suggested the expression of *Ca*. Eudoremicrobiaceae polyunsaturated hydrocarbon biosynthetic cluster is constitutive. This finding is consistent with previous studies that have shown that transcription of PUFA genes in the deep-sea bacterium *Photobacterium profundum* strain SS9 does not change under variable cultivation conditions such as increased pressure or reduced

temperature despite an observed increase in PUFA production[138]. An independent study found that hydrocarbons produced via the *oleABCD* pathway are also constitutively produced[139].

Ca. Eudoremicrobium-*specific biosynthetic potential*

Despite this shared biosynthetic potential, the *Ca.* Eudoremicrobium genus stood out as its members were found to encode for up to 15 additional BGCs. Among them, 10 were identified as NRPS and PKS clusters and seven as RiPPs spanning multiple peptide classes[140], including proteusin pathways predicted by antiSMASH, more than any organism characterized so far. Proteusins are of particular biotechnological interest owing to their varied bioactivities and the expected density and diversity of unusual chemical modifications installed by enzymes encoded in relatively short BGCs[45].

T1PKS/3*NRPS

Notably, *Ca.* Eudoremicrobium representatives encode for a particularly large (over 70 kbp) and architecturally complex type I PKS/NRPS hybrid. An unusual feature of this cluster is the presence of terminal reductase domains in both of the two NRPS proteins. Notably, the majority of characterized natural products modified by these domains have shown varied and enhanced bioactivity such as protease inhibition and antitumoral properties[141].

More specifically this pathway contains a hybrid T1PKS/NRPS megasynth(et)ase with two neighboring NRPS modules forming an interleaved cluster 77 kb in length (Figure 3D). The total of four adenylation domains in the cluster were all predicted to have specificity for aromatic amino acids such as phenylalanine, tryptophan, hydroxyphenylglycine or dihydroxybenzoate by NRPSsp[142]. The BGC shares some similarities with the bacilysin pathway found in *Bacillus* spp.[143] Bacilysin is a dipeptide 'Trojan horse' antibiotic in which L-alanine is bound to L-anticapsin for export prior to peptidase cleavage and release of the active anticapsin drug. Both pathways encode relatively small peptidic products with aromatic side chains that are highly modified by an abundance of reductases and dehydrogenases in the cluster (Extended Data Fig. 8a). However, unlike the bacilysin pathway which produces a dipeptide, we predict the *Ca*. Eudoremicrobiaceae BGC encodes a hybrid PKS/NRPS final product, which is likely also glycosylated as suggested by the presence of two glycosyltransferases encoded in the cluster. Additional Fe(II)/α-ketoglutarate-dependent oxygenases and *O*-demethylase Rieske oxygenases suggests the final product is likely more oxidized than bacilysin.

Candidate proteusin BGC 54.1

The most complex, lineage-specific RiPP BGC identified in *Ca*. Eudoremicrobium BGC is of exceptional intricacy as it encodes over 10 maturation enzymes, including a lanthionine synthetase and three epimerases that likely modify a precursor peptide along with the complete genetic machinery for cleavage and transport of the mature peptide (Extended Data Fig. 8b). The pathway is classified as a proteusin by antiSMASH and the predicted presence of D-amino acids and (methyl)lanthionine rings resulting from the first half of the cluster suggests some shared structural features to the recently characterized antiviral landornamides[46]. However, the precursor peptide appears to be similar to Nif11-type leaders

as opposed to the usual NHLP leader of proteusins, suggesting that it could be a non-proteusin epimerized RiPP.

More specifically, the precursor peptide contains a Nif11-like leader portion, with a canonical C-terminal Gly-Gly cleavage motif. Notably, a second Gly-Gly site is identified within the predicted core region. A LanM-type lanthionine synthetase (encoded by *orf9*) putatively installs up to four lanthionine bridges on the precursor peptide, as four cysteine residues are present in the core peptide. Predicted radical SAM (rSAM) epimerases (*orf10, orf11, orf19*) homologous to PoyD and OspD are likely to install ᴅ-amino acids on the peptide, and B_{12}-dependent radical SAM enzymes (*orf12*, *orf21*) homologous to C-methyltransferases potentially methylate carbons in the precursor[45,92]. A number of other metalloenzymes belonging to the rSAM, P450, mononuclear non-heme iron α-ketoglutarate dependent families, encoded by *orf13*, *orf14*, *orf16*, *orf17*, *orf20*, likely oxidize the peptide product. Finally, predicted transporters (*orf3*-*orf7*), including some with an N-terminal C39 peptidase, are expected to both cleave the Nif11-type leader peptide and export the mature natural product to the extracellular milieu. This cluster is also co-localized with an uncharacterized family of phage plasmid transfer proteins (encoded by *orf22*) found in the plasmid SCP1 of *Streptomyces coelicolor* and various *Mycobacterium* phage genomes, suggestive of cluster mobility.

Proteusin BGC 34.1

This proteusin cluster contains a precursor peptide rich in small hydrophobic amino acids and harboring an NX_5N pattern of residues in the core region. Together with the presence of other key maturases such as rSAM epimerases (Extended Data Fig. 8c), this cluster is highly characteristic of pore-forming β-helix peptides, such as the highly cytotoxic polytheonamides and aeronamides[45,89,144]

Deep-sea specific BGCs

Aryl polyene

The deep-sea *Ca*. Eudoremicrobium (*Ca*. E malaspinii) also have a unique aryl polyene cluster, which is surprising given that aryl polyenes typically play a role in protection from photodamage by visible light by quenching reactive oxygen species (ROS). However, visible light does not reach depths of 2,000 – 4,000 m where organisms with the aryl polyene cluster are exclusively found. This suggests the aryl polyene product might play a different ecological role, or that ROS may be generated from other sources. A recent study found that marine microbial ROS production through one-electron reduction of O_2 to superoxide production plays a larger role in the marine oxygen cycle than previously realized[123]. Indeed, ‘dark’ biological superoxide production was demonstrated in most major groups of marine microbes. This suggests ROS mitigation strategies such as through aryl polyene production may also be essential for the survival of bathypelagic microbes even in the absence of visible light.

RiPP BGC 75.1

Additionally, we found the deep ocean species (*Ca*. E. malaspinii) representatives to encode for a unique RiPP cluster within these clades. Due to this particularity, we selected this cluster for further characterization.

Ca. Eudoremicrobiaceae as a source of new enzymes and natural products

For experimental validation of biosynthetic pathways in *Ca*. Eudoremicrobiaceae we selected the best short-read genome of the type species (*Ca*. E. malaspinii), i.e. the MAG MALA_SAMN05422137_METAG_HLLJDLBE, as the reference genetic sequence.

Experimental validation of Ca. E. malaspinii *RiPP BGC 75.1*

In silico cluster analysis

Protein sequence similarity[145] shows EmbA harbors an N-terminal leader region commonly found in ribosomal natural products, with homology to the Nif11 enzyme, involved in nitrogen fixation. The C-terminus of the leader peptide includes the prototypical Gly-Gly cleavage motif, where the leader peptide is removed for complete maturation of the peptide product, resulting in an 18 amino acid core with sequence MVTTFFIPSESDDQFFKK. Using the DeepRiPP workflow, EmbA is predicted to be a class II lanthipeptide with 79% class prediction probability, but very low similarity to other RiPP cores[146]. Threonine and serine residues are present in the core sequence, and could be sites for phosphorylation and dehydration, as is characteristic in lanthipeptide biosynthesis. Nonetheless, cysteines are not found in the core peptide sequence, eliminating the possibility of (methyl)lanthionine macrocycle formation. BLASTp analysis on EmbM shows homology to the dehydration domain of type 2 lanthipeptide biosynthesis proteins, generally termed LanM, which are bifunctional enzymes capable of catalyzing dehydration and cyclization reactions on their substrates[147]. EmbM was modelled using Phyre2[148]. In accordance with BLAST results, EmbM shows predicted structural similarity to protein kinases and to the dehydratase domain of CylM (83% coverage of the sequence, modelled with 100% confidence), the Enterococcal lanthipeptide synthetase enzyme from the cytolysin biosynthetic pathway (Extended Data Fig. 9a-b)[43]. EmbM, however, does not contain the typical Zn-dependent cyclization domain which catalyzes the lanthionine macrocycle formation between the thiol side chain of Cys and dehydroamino acids. This observation is consistent with the lack of cysteine residues in the precursor peptide EmbA. Notably, biosynthetic enzymes homologous to the dehydration domain of LanM and lacking the cyclization domain are also present in the biosynthetic clusters for the polytheonamides and aeronamides, highly cytotoxic compounds from '*Candidatus* Entotheonella factor' and *Microvirgula aerodenitrificans*[45,89,149]. These enzymes, PoyF and AerF, were shown to dehydrate threonines at core position 1.

In CylM, residues required for phosphorylation are Lys274, Asp347, His349, Asn352, and Asp364[43]. In EmbM, sequence alignment and structural modelling allows us to map these residues to Lys178, Asp252, His254, Asn257, and Asp269, suggesting EmbM's ability to phosphorylate Thr or Ser residues in the precursor peptide core (Extended Data Fig. 9c-d). Mutagenesis investigations in representative lanthipeptide synthetases and CylM have identified residues Lys274, Asp252, His254, Arg506 and Thr512 as key for facilitating phosphate elimination[43,147,150,151]. Lys274, Asp252 and His254 are proposed to both activate the phosphate group for transfer from ATP and stabilize the phosphate in the elimination step. In EmbM, the first two residues correspond to Lys178 and Asp155. Notably, EmbM is missing the equivalent of His254, and this residue corresponds to a Leu or a Pro in EmbM based on structure and sequence comparisons, respectively. In CylM, mutational analysis showed that

a CylM-H254A mutant completely abolished phosphate elimination and generation of dehydrated products.

Arg506 and Thr512 are proposed to directly assist phosphate elimination in CylM. Thr512 is thought to act as a general base to deprotonate the α-carbon of the phosphorylated residue, generating an enolate, which is stabilized by the side chain of Arg506. In EmbM, CylM's Arg506 is mapped to Arg390 and CylM's Thr512 to Ser396 (Extended Data Fig. 9c-d). Although highly unusual, Thr to Ser mutation is not unprecedented: in *Nostoc sp.* 106C, a LanM enzyme (WP_086758087) with a Ser as predicted general base is associated with a Nif11 precursor peptide (WP_086758085). To our knowledge, the function of this particular LanM enzyme has not been experimentally validated. Nonetheless, mutagenesis studies in CylM indicate that a T512A mutant is capable of generating phosphorylated intermediates, but elimination and generation of dehydrated peptides is not observed[43].

Immediately downstream of *embM* is *orf3*. The resulting small protein, which we termed EmbI, shows only distant similarity to proteins of unknown function. Notably, EmbI shows no similarity to PqqD, the prototypical RiPP recognition element (RRE)[152,153]. A prediction of secondary structural elements of EmbI using PSIPRED and analysis using transmembrane hidden Markov model (TMHHM), indicates the EmbI is comprised of two alpha helices, of which the C-terminal is predicted to be a transmembrane helix[154,155]. Along with the high calculated isoelectric point and proximity to bacteriocin biosynthetic genes, an immunity related function is suggested for EmbI. The scaffold containing *embA* and *embM* also harbors open reading frames likely involved in regulation via a two-component system. Orf1 is predicted to act as a sensory histidine kinase and Orf2 shows high homology to response regulators. The C-terminus of Orf6 shows distant homology to peptidase family C40, often involved in cell wall degradation or remodeling[156]. Finally, Orf7 shows homology to glutamine aminotransferase, which takes part in the biosynthesis of guanosine nucleotides. Typical transporters associated with Nif11-type precursor peptides are not present in the cluster neighborhood.

Functional characterization

In order to functionally characterize cluster 75.1, heterologous co-expression experiments and *in vitro* enzymatic assays were conducted. Heterologous co-expression in *E. coli* was initially performed with the constructs pACYCDuet-1-EmbA(MCS1) in *E. coli* BL21(DE3), as a negative control; and pACYCDuet-1-EmbA(MCS1) + pCDFDuet-1-EmbM(MCS2) in *E. coli* BL21(DE3) (Supplementary Table 6). Expression of both *embA* and *embM*, followed by nickel-affinity chromatography for purification of EmbA resulted in high expression levels for both proteins. Proteolysis with trypsin followed by HPLC-MS analysis resulted in the appearance of two new peaks in the chromatogram upon comparison with an experiment in which only EmbA was produced. The new peaks displayed monoisotopic masses $(M+3H)^{3+}$ 1182.8769 and $(M+3H)^{3+}$ 1209.5277, which correspond to a difference of +79.9743 and and +159.9267, respectively, in relation to the unmodified trypsin digest fragment of EmbA (Supplementary Table 6). This mass difference corresponds to the single and double phosphorylation of EmbA (monophosphorylated EmbA calculated mass $(M+3H)^{3+}$ 1182.8685; double phosphorylated EmbA calculated mass $(M+3H)^{3+}$ 1209.5239, Supplementary Table 6). HPLC-MS/MS was utilized to localize the modifications in the peptide sequence. Fragment ions y16, y15, y14 and

b17, b16, b15 allow us to map modifications to Thr(3) and Thr(4) for monophosphorylated products and both Thr(3) and Thr(4) for doubly phosphorylated products (Extended Data Fig. 9j, Supplementary Table 6). Fragment ions corresponding to Thr(4) phosphorylation were more abundant, pointing to initial modification at that position being favored. Cleavage of EmbA with LahT150[90] resulted in the removal of the leader peptide and identification of mono and double phosphorylated peptide cores, in line with the results obtained with trypsin (Supplementary Table 6).

In silico analysis of EmbM suggests the enzyme is capable of phosphorylation of substrate peptides, given all residues proposed to be involved in kinase activity of CylM can be mapped to EmbM. This hypothesis is confirmed experimentally with the results described above. EmbM is generally similar to AerF, PoyF and the dehydration domain of LanM enzymes. However, EmbM does not contain key active site residues that are responsible for catalyzing phosphate elimination, and we hypothesized that the linear, phosphorylated peptide could be the terminal product of the EmbM maturase. Our hypothesis was supported by the fact that no dehydrated EmbA was found in the initial experiments described above. A number of additional co-expression conditions were tested: *E. coli* expression hosts were varied, along with expression media, temperature, and time (Supplementary Table 6). Different construct combinations were also tested: precursor and EmbM were expressed in their genetic context or expressed from individual plasmids with different copy numbers and the genes encoding each protein were also codon-optimized for *E. coli* expression. EmbI was also included in co-expression experiments, given its putative role in providing the native producing organism, and likely the heterologous expression host, with immunity to the biological activity of the cluster product. All conditions assayed resulted in the same outcome: high amounts of phosphorylated products were detected, but dehydration was not observed.

Co-expression of EmbA and EmbM was also performed in *Microvirgula aerodenitrificans*, a Betaproteobacterium isolated from activated sludge[89]. We hypothesized that, due to the broad similarities between EmbM and AerF and the fact that *M. aerodenitrificans* is also found in aquatic environments, EmbM was likely to display its full predicted activity. Nonetheless, 94% of the precursor peptide was converted to phosphorylated products (percent conversion was calculated based on the relative peak areas in extracted ion chromatograms). *In vitro* enzymatic assays with EmbA and EmbM were also conducted. The proteins were individually purified and assayed with $MgCl_2$ and ATP as a co-substrate. A variety of conditions were screened and, upon incubation of EmbA and EmbM at 37 °C for 3 days, upwards of 75% conversion to phosphorylated products was observed, in line with the results of co-expression experiments.

Intriguingly, efficient phosphorylation by wild-type LanM-type enzymes without concomitant β-elimination and generation of dehydrated amino acids is, to our knowledge, unprecedented. We attribute this result to the fact that EmbM is missing one of the three residues thought to stabilize the phosphate group for elimination and, notably, the predicted base that deprotonates the α-carbon of phosphorylated residues is a Ser as opposed to a more common Thr. This is supported by the consistency of the mutations across all MAGs containing this BGC. In the model enzyme CylM, the equivalent residues (H254, T512) were shown to be essential for phosphate elimination based on mutational analysis[43].

In order to test and compare the biological activities of precursor peptide, phosphorylated peptide and hypothetically dehydrated peptide, dehydration of phosphorylated peptide was recreated *in vitro* by combining enzymatic and chemical methods. Single and double phosphorylated products were generated by reaction of EmbA and EmbM. Cleavage of the leader peptide with the promiscuous LahT150 protease yielded the phosphorylated core peptide. Finally, β-elimination of phosphorylated peptides was achieved by treatment with base. Production of single and double dehydrated products was confirmed by HR-MS and HR-MS/MS (Supplementary Table 6, Extended Data Fig. 9k). Further confirmation of the presence of α,β-unsaturated Thr residues resulted from derivatization of EmbA trypsin fragments with DTT (Supplementary Table 6, Extended Data Fig. 9k). Dehydrobutyrine residues are more stable in the *Z* configuration and, with the exception of cypemycin, all Dhb-containing RiPPs harbor the *Z* isomer[157–159]. We thus depict Dhb(3) and Dhb(4) in the *Z* configuration.

A number of biological activity screens were performed with unmodified precursor peptide, phosphorylated peptide and dehydrated peptide. MTT assays resulted in no cytotoxic effect at biologically-relevant concentrations against HeLa cells for all peptides. Similarly, no antimicrobial activity was observed against a varied panel of bacteria. Protease inhibition screens yielded no activity against trypsin, chymotrypsin and cathepsin B. Gratifyingly, however, the phosphorylated peptide inhibited neutrophil elastase at an IC_{50} of 14.3 uM, whereas the precursor and dehydrated peptides showed no activity.

In silico analysis and functional characterization of EreA

Through our analysis of the *Ca.* Eudoremicrobium biosynthetic potential, we identified a BGC that was highly-expressed (Figure 3D) and predicted by antiSMASH to produce a RiPP belonging to the proteusin class of natural products[160]. The 5.2-kb BGC, *ereAIMDB*, encodes a nitrile hydratase-like precursor (NHLP) and four candidate maturases (Figure 4E). In comparison to the polytheonamide BGC, the locus did not encode a PoyE-like asparagine-N-methyltransferase homolog nor did the core sequence have interlocking ‘NX_5N’ motifs critical for the β-helical peptide secondary structure[161] of the pore-forming polytheonamide-like RiPPs[89,149]. Instead, the EreA 46 aa core consisted mainly of hydrophobic residues including a 4x repeating ‘GGP[T/S]’ motif and 16 valines residues. The protein encoded by the full-length 406-bp *ereA* precursor gene contains a characteristic ‘AVAGG’ RiPP cleavage motif preceded by a sequence of acidic residues, which we predicted would enable proteolysis by the promiscuous RiPP peptidase LahT150[90]. Heterologous expression of the *Nhis-ereA* precursor gene in *E. coli* BL21(DE3) followed by purification, proteolytic digest with LahT150, and HPLC-HR-MS/MS analysis resulted in identification of a chromatogram peak with a $(M+3H)^{3+}$ of 1433.1095 Da corresponding to the 46 aa unmodified core peptide (Extended Data Fig. 11a). We next co-expressed each maturase gene in the BGC in combination with the *Nhis-ereA* precursor gene.

EreI: Aspartinyl-asparaginyl β-hydroxylase protein family

The most upstream predicted maturase encoded in the cluster, EreI, is an iron(II)/2-oxoglutarate-dependent oxygenase belonging to the aspartinyl/asparaginyl β-hydroxylase protein family. EreI shares 22% aa identity with PoyI, a hydroxylase in the polytheonamide BGC, the first characterized member of the proteusin RiPP class[149]. *In vivo* modification was

not observed when *ereI* was expressed solely with *Nhis-ereA*. *In vitro* assays with purified NHis-EreI also failed to modify the EreA or EreA + EreD core. In contrast, when *Nhis-ereA* was co-expressed in combination with other maturases, *ereIMD* and *ereIMDB*, peptides with a mass shift corresponding to the addition of one methyl group and incorporation of one oxygen was detected (Extended Data Fig. 11a). The oxygen incorporation could only be localized to the two C-terminal residues of the core by MS^2 analysis (Extended Data Fig. 11b). The most likely position is at a *tert*-Leu methyl group, since hydroxylations at any other site would result in an unstable molecule.

EreM: New RiPP maturase in the FkbM-like methyltransferase family

EreM is a new predicted type of RiPP maturase belonging to the FkbM-like *O*-methyltransferase family. *In vivo* co-expressions of *ereM* with *Nhis-ereA* resulted in a new peak in the HPLC-HRMS chromatogram compared to experiments in which only *NHis-ereA* was expressed. The new peak displayed a monoisotopic mass of $(M+3H)^{3+}$ 1437.7845 corresponding to the addition of CH_2 (+14.0143 Da) in comparison to the unmodified LahT-digested EreA core (Extended Data Fig. 10b). FkbM-like enzymes are *S*-adenosyl methionine (SAM) dependent, therefore we set up *in vitro* enzyme cascades to biosynthesize either SAM or ^{13}C-SAM, respectively[162]. *In vitro* reactions indicated a single methyl group (14.0143 Da) or ^{13}C-methyl group (15.0146 Da) was incorporated (Extended Data Fig. 10a) and detected in the corresponding 48 aa long N-terminally Gly-Gly-extended EreA core fragments with the monoisotopic masses of $(M+3H)^{3+}$ 1475.7971 Da and 1476.1310 Da, respectively. MS^2-fragmentation of the two methylated 48mer EreA core fragments suggested methylation at the terminal cysteine residue (Extended Data Fig. 10b, Supplementary Table 5). In order to elucidate which heteroatom is methylated by EreM, we subsequently performed HSQC NMR spectroscopy on an epimerized product of Nhis-EreA + EreM *in vitro* assay using ^{13}C-labeled $^{13}CH_3$-L-methionine. The HSQC spectrum revealed two signals with chemical shifts of 2.03/17.3 ppm and 2.88/25.9 ppm (Extended Data Fig. 10d) that we assumed as ^{13}C-heteroatom bonds based on chemical shifts. Using C-H-uncoupled ^{1}H NMR analysis and comparison to a standard ^{1}H NMR, our assumption was supported by observation of peak splitting indicating ^{13}C-H bonds of ^{13}C-labeled methyl groups (Extended Data Fig. 10c). In addition, four signals with chemical shifts of 3.46/70.0 ppm, 3.55/70.0 ppm, 3.64/62.2 ppm and 3.69/74.6 ppm were detected. The detected signals were further assigned by comparison with the literature: The four downfield signals are most likely from the Tris buffer[108]. Comparison of the chemical shifts with the NMR assays performed by Mordhorst et al. suggested that the signal at 2.88/25.9 ppm shows a ^{13}C-N bond and the signal at 2.03/17.3 ppm shows a ^{13}C-S bond. The latter is of residual $^{13}CH_3$-L-methionine origin from the *in vitro* assay. Based on the observed MS^2-fragments (Extended Data Fig. 10b) the ^{13}C-N bond would be part of the valine-cysteine amide moiety. The formation of an *O*-methylated iminol tautomer of the amide bond was excluded due to the higher chemical shifts reported for model compounds of the two tautomers (~1 ppm in ^{1}H NMR, ~15 ppm in ^{13}C NMR)[105,106]. Comparison with chemical shifts of *N*-methylated amide bonds in omphalotin further supported the ^{13}C-N bond assignment (chemical shifts in omphalotin - valine: 2.71-2.97 ppm for ^{1}H NMR, 28.6-29.8 ppm for ^{13}C NMR; isoleucine: 2.59 and 3.01 ppm for ^{1}H NMR, 29.1 and 30.5 ppm for ^{13}C NMR; and glycine: 2.81-2.93 ppm for ^{1}H NMR, 36.0-36.3 ppm for ^{13}C NMR)[109]. *S*-methylation of the cysteine side chain

was excluded by the chemical shifts of *N*-acetyl-*S*-methyl-DL-cysteine (2.11 ppm for ^1H NMR, 17.7 ppm for ^{13}C NMR)[107]. Regarding the reported chemical shift and the detected signal at 2.03/17.3 ppm, a mixture of *N*- and *S*-methylated cysteine cannot be completely ruled out. However, the presence of an additional signal at 2.88/25.9 ppm makes *S*-methylation of cysteine less likely. To our knowledge, this peptide bond *N*-methylation is the first report of *N*-methyltransferase activity by an FkbM-like *O*-methyltransferase family enzyme and first demonstration of an involvement in RiPP modification.

EreM shares 28% aa identity with FkbM from *Streptomyces hygroscopicus* (AAF86398.1), the namesake for the protein family. FkbM transfers a methyl group from SAM to the 31-O position of the immunosuppressant polyketide FK506[47,163]. To compare the characterized activity of EreM to FkbM-like methyltransferases in other biosynthetic pathways, we searched all BGCs in MIBiG v 2.0 using the standard FkbM-like methyltransferase family Hidden Markov Model (HMM, Methyltransf_21, PF05050) and identified 29 hits within characterized BGC boundaries (Extended Data Fig. 10e). Four of the 29 FkbM homologs had been previously functionally characterized either by genetic knockout studies or heterologous expression and all were found to be *O*-methyltransferases. Based on biosynthetic logic and the final natural product structure, the remaining 25 hits in MIBiG were also proposed in the literature to be *O*-methyltransferases, suggesting that EreM is the first FkbM family member to display *N*-methyltransferase activity. Moreover, none of the FkbM hits were in RiPP biosynthetic pathways, further supporting that peptide bond *N*-methylation by EreM is, to the best of our knowledge, a new role of FkbM members in posttranslational modification. Additionally, we did not find any report of N-methyltransferase activity for any member of the FkbM family.

To probe the substrate range of EreM, we tested for *in vivo* activity with a library of core variants ranging between 35 and 46 aa in length (Extended Data Fig. 10f). We observed *in vivo* incorporation of a single methyl group by EreM for all core analogs tested (Extended Data Fig. 10g), which was localized to the C-terminus of the peptide (data not shown). Moreover, when *Nhis-ereA* was co-expressed with a subset of other maturase genes, i.e., *ereIMD*, we observed mass shifts corresponding to up to six methylations per core peptide (Extended Data Fig. 10h), whereas in constructs lacking *ereI* we only observed a single methylation (Extended Data Fig. 10g). Multiple methylation sites could be localized by MS^2 analysis to V9, V13, V15, V37, V44, and V45/C46 (Extended Data Fig. 10i). No hydroxylations were observed at these residues, suggesting that amide bonds were the sites of methylation. This multiple methylation pattern observed in conjunction with EreI suggests protein-protein interactions between EreM and EreI may be necessary for multiple methylations to occur. Such protein-complex formation was proposed earlier for RiPP maturation in lipolanthine biosynthesis where allosteric activation of a precursor-maturase complex by an additional enzyme may be required[164]. EreM activity with a variety of core analogs opens new areas of research to explore the biotechnological potential of EreM to generate mono- and multi-methylated peptides.

EreD: radical SAM epimerase

We next tested the activity of the radical SAM epimerase EreD. Co-expression of *Nhis-ereA* and *ereD* in *E. coli* resulted in a product with an identical *m/z* to the unmodified core but improved product solubility and a 0.47 minute retention time shift relative to the unmodified core suggesting epimerization (Extended Data Fig. 11c). Analysis using an orthogonal D_2O-

based induction system (ODIS)[165], which incorporates a deuterium atom at each epimerized site that can be localized by MS^2, resulted in deuteration of seven residues (Figure 19B), five valines and two alanines (V10, A12, A14, V16, V18, V29, and either V44 or V45). Weak MS^2-fragmentation did not allow us to distinguish the epimerization site between the final two C-terminal valine residues, although a mass shift corresponding to one epimerized residue was detected in this region. Advanced Marfey's analysis of the peptide confirmed the presence of D-Val and D-Ala residues, in a ratio with L-Val and L-Ala, respectively of approximately 1:3 (Extended Data Fig. 11f). Based on the core amino acid composition, this would correspond to five valines and two alanines and a total of seven epimerized residues as confirmed by a mass shift of +7.04 Da by ODIS (Extended Data Fig. 11e). The first five epimerized sites display an alternating pattern similar to previously reported epimerase activities such as PoyD[149] which shares 36% aa identity with EreD, while the remaining peptide portion features a distinct epimerization pattern.

EreB: B_{12}-dependent C-methyltransferase active in a non-standard heterologous host

We observed no activity of the B_{12}-dependent C-methyltransferase, EreB, in *E. coli,* even when co-expressed with a complete BtuCEDFB cobalamin uptake system and with added hydroxycobalamin, $FeCl_3$, and cysteine[166]. Recently, we developed the betaproteobacterial wastewater denitrifier, *Microvirgula aerodenitrificans* containing a complete pathway for cobalamin biosynthesis as an alternative heterologous expression platform for B_{12}-dependent RiPP maturases[89]. We cloned *Nhis-ereAD*, *Nhis-ereADB, Nhis-ereAIMD, and Nhis-ereAIMDB* into the pLMB509-m vector (an arabinose-inducible variant of pLMB509) and conjugated the constructs individually into the newly-constructed *M. aerodentrificans Δaer*, which carries a deletion of the native aeronamide RiPP BGC[89] containing a B_{12}-dependent C-methyltransferase gene. Following successful conjugation, expression, and purification of the EreA core from *M. aerodenitrificans Δaer*, we observed the formation of a mixture of multiple methylated products absent in our control constructs lacking *ereB.* We detected mass shifts up to +98.13 Da corresponding to the addition of 7 methyl groups (Extended Data Fig. 12a). Following proteolytic digestion and MS^2 analysis, we were able to localize C-methylation sites to seven different valine residues in the core peptide: V9, V13, V15, V35, V37, V44, and V45 (Extended Data Fig. 12c). Similar to reports from B_{12}-dependent C-methylation of the polytheonamides, we always obtained a mixture of C-methylated products (Extended Data Fig. 12b) despite testing a range of expression conditions (Supplementary Table 5). Evidence for side chain C-methylation of valines was further supported by advanced Marfey's detection of *tert*-Leu residues in cores purified from *M. aerodentrificans* (Extended Data Fig. 12d).

Defining BGC boundaries

Through comparative BGC analysis, close homologs of the four maturase genes *ereIMDB*, were co-localized with a highly similar NHLP-like precursor conserved across all *Ca.* Eudoremicrobium MAGs. To test the BGC boundaries, we cloned and co-expressed three flanking ORFs downstream from *ereAIMDB* from *Ca.* Eudoremicrobium malaspinii. EreF was annotated as a homocysteine *S*-methyltransferase and EreG as a carboxymuconolactone decarboxylase family enzyme. EreR shares homology to IscR transcription factors that regulate Fe-S cluster biogenesis[167]. We did not observe any effects or additional core modifications from multiple co-expressions of *Nhis-ereA*, *Nhis-ereAD,* and *Nhis-ereAID* with

ereF, *ereR*, or *ereG*. Based on these findings, we propose *ereAIMDB* forms the complete proteusin BGC. In conclusion, despite its relatively small size (5.2-kbp) the BGC characterized here encodes remarkable biosynthetic complexity. Based on our experimental characterization of the four RiPP maturases, up to 1 hydroxylation, 7 epimerizations, and 13 methylations are introduced into a 46 aa core (Figure 4G).

Supplementary Table Legends

Supplementary Table 1

Accession numbers and metadata for samples, genomes, metagenomes, metatranscriptomes, assemblies, bins and MAGs used in this study.

Supplementary Table 2

Data associated with describing the biosynthetic potential of the ocean microbiome, including the clustering of BGCs into GCFs and GCCs, and its metagenomic structuring.

Supplementary Table 3

Information about BGC-rich lineages described in this study with details on the *Ca*. Eudoremicrobiaceae family, such as taxonomy and biosynthetic potential annotations.

Supplementary Table 4

Supplementary information and data for the transcriptomic analysis of natural *Ca*. E. taraoceanii populations, including detailed genome annotations and differentially expressed functions.

Supplementary Table 5

Supplementary information and data for the experimental characterization of the pythonamide pathway.

Supplementary Table 6

Supplementary information and data for the experimental characterization of the phospeptin pathway.

References

1. Hug, L. A. *et al.* A new view of the tree of life. *Nature Microbiology* vol. 1 (2016).
2. Newman, D. J. & Cragg, G. M. Natural Products as Sources of New Drugs over the Nearly Four Decades from 01/1981 to 09/2019. *J. Nat. Prod.* **83**, 770–803 (2020).
3. Adrio, J. L. & Demain, A. L. Microbial enzymes: tools for biotechnological processes. *Biomolecules* **4**, 117–139 (2014).
4. Medema, M. H., de Rond, T. & Moore, B. S. Mining genomes to illuminate the specialized chemistry of life. *Nat. Rev. Genet.* (2021).
5. Cavicchioli, R. *et al.* Scientists' warning to humanity: microorganisms and climate change. *Nat. Rev. Microbiol.* **17**, 569–586 (2019).
6. Pye, C. R., Bertin, M. J., Lokey, R. S., Gerwick, W. H. & Linington, R. G. Retrospective analysis of natural products provides insights for future discovery trends. *Proc. Natl. Acad. Sci. U. S. A.* **114**, 5601–5606 (2017).
7. Davies, J. Specialized microbial metabolites: functions and origins. *J. Antibiot.* **66**, 361–364 (2013).
8. Lloyd, K. G., Steen, A. D., Ladau, J., Yin, J. & Crosby, L. Phylogenetically Novel Uncultured Microbial Cells Dominate Earth Microbiomes. *mSystems* **3**, (2018).
9. Robinson, S. L., Piel, J. & Sunagawa, S. A roadmap for metagenomic enzyme discovery. *Nat. Prod. Rep.* (2021).
10. Venter, J. C. *et al.* Environmental genome shotgun sequencing of the Sargasso Sea. *Science* **304**, 66–74 (2004).
11. Qin, J. *et al.* A human gut microbial gene catalogue established by metagenomic sequencing. *Nature* **464**, 59–65 (2010).
12. Sunagawa, S. *et al.* Structure and function of the global ocean microbiome. *Science* **348**, (2015).
13. Parks, D. H. *et al.* A standardized bacterial taxonomy based on genome phylogeny substantially revises the tree of life. *Nat. Biotechnol.* **36**, 996–1004 (2018).
14. Nayfach, S., Rodriguez-Mueller, B., Garud, N. & Pollard, K. S. An integrated metagenomics pipeline for strain profiling reveals novel patterns of bacterial transmission and biogeography. *Genome Res.* **26**, 1612–1625 (2016).
15. Crits-Christoph, A., Diamond, S., Butterfield, C. N., Thomas, B. C. & Banfield, J. F. Novel soil bacteria possess diverse genes for secondary metabolite biosynthesis. *Nature* vol. 558 440–444 (2018).
16. Nayfach, S. *et al.* A genomic catalog of Earth's microbiomes. *Nat. Biotechnol.* 1–11 (2020).

17. Zan, J. *et al.* A microbial factory for defensive kahalalides in a tripartite marine symbiosis. *Science* **364**, (2019).

18. Wilson, M. C. *et al.* An environmental bacterial taxon with a large and distinct metabolic repertoire. *Nature* **506**, 58–62 (2014).

19. Pasolli, E. *et al.* Extensive Unexplored Human Microbiome Diversity Revealed by Over 150,000 Genomes from Metagenomes Spanning Age, Geography, and Lifestyle. *Cell* **176**, 649–662.e20 (2019).

20. Pachiadaki, M. G. *et al.* Charting the Complexity of the Marine Microbiome through Single-Cell Genomics. *Cell* **179**, 1623–1635.e11 (2019).

21. Biller, S. J. *et al.* Marine microbial metagenomes sampled across space and time. *Sci Data* **5**, 180176 (2018).

22. Salazar, G. *et al.* Gene Expression Changes and Community Turnover Differentially Shape the Global Ocean Metatranscriptome. *Cell* **179**, 1068–1083.e21 (2019).

23. Acinas, S. G. *et al.* Deep ocean metagenomes provide insight into the metabolic architecture of bathypelagic microbial communities. *Commun Biol* **4**, 604 (2021).

24. Alneberg, J. *et al.* Binning metagenomic contigs by coverage and composition. *Nat. Methods* **11**, 1144–1146 (2014).

25. Parks, D. H. *et al.* Recovery of nearly 8,000 metagenome-assembled genomes substantially expands the tree of life. *Nat Microbiol* **2**, 1533–1542 (2017).

26. Delmont, T. O. *et al.* Nitrogen-fixing populations of Planctomycetes and Proteobacteria are abundant in surface ocean metagenomes. *Nat Microbiol* **3**, 804–813 (2018).

27. Klemetsen, T. *et al.* The MAR databases: development and implementation of databases specific for marine metagenomics. *Nucleic Acids Res.* **46**, D692–D699 (2018).

28. Jain, C., Rodriguez-R, L. M., Phillippy, A. M., Konstantinidis, K. T. & Aluru, S. High throughput ANI analysis of 90K prokaryotic genomes reveals clear species boundaries. *Nat. Commun.* **9**, 5114 (2018).

29. Kautsar, S. A., Blin, K., Shaw, S., Weber, T. & Medema, M. H. BiG-FAM: the biosynthetic gene cluster families database. *Nucleic Acids Res.* **49**, D490–D497 (2021).

30. Kautsar, S. A. *et al.* MIBiG 2.0: a repository for biosynthetic gene clusters of known function. *Nucleic Acids Res.* **48**, D454–D458 (2020).

31. Klassen, J. L. & Currie, C. R. Gene fragmentation in bacterial draft genomes: extent, consequences and mitigation. *BMC Genomics* **13**, 14 (2012).

32. Timmermans, M. L., Paudel, Y. P. & Ross, A. C. Investigating the Biosynthesis of

Natural Products from Marine Proteobacteria: A Survey of Molecules and Strategies. *Mar. Drugs* **15**, (2017).

33. Shah, S. A. A. *et al.* Structural Diversity, Biological Properties and Applications of Natural Products from Cyanobacteria. A Review. *Mar. Drugs* **15**, (2017).

34. Wiegand, S. *et al.* Cultivation and functional characterization of 79 planctomycetes uncovers their unique biology. *Nature Microbiology* vol. 5 126–140 (2020).

35. Ceniceros, A., Dijkhuizen, L., Petrusma, M. & Medema, M. H. Genome-based exploration of the specialized metabolic capacities of the genus Rhodococcus. *BMC Genomics* **18**, 593 (2017).

36. Gregory, K., Salvador, L. A., Akbar, S., Adaikpoh, B. I. & Stevens, D. C. Survey of Biosynthetic Gene Clusters from Sequenced Myxobacteria Reveals Unexplored Biosynthetic Potential. *Microorganisms* **7**, (2019).

37. Woodcroft, B. J. *et al.* Genome-centric view of carbon processing in thawing permafrost. *Nature* **560**, 49–54 (2018).

38. Ward, L. M., Cardona, T. & Holland-Moritz, H. Evolutionary Implications of Anoxygenic Phototrophy in the Bacterial Phylum Eremiobacterota (WPS-2). *Front. Microbiol.* **10**, 1658 (2019).

39. Ji, M. *et al.* Candidatus Eremiobacterota, a metabolically and phylogenetically diverse terrestrial phylum with acid-tolerant adaptations. *ISME J.* (2021) doi:10.1038/s41396-021-00944-8.

40. Sunagawa, S. *et al.* Tara Oceans: towards global ocean ecosystems biology. *Nature Reviews Microbiology* vol. 18 428–445 (2020).

41. Pérez, J., Moraleda-Muñoz, A., Marcos-Torres, F. J. & Muñoz-Dorado, J. Bacterial predation: 75 years and counting! *Environ. Microbiol.* **18**, 766–779 (2016).

42. Arnison, P. G. *et al.* Ribosomally synthesized and post-translationally modified peptide natural products: overview and recommendations for a universal nomenclature. *Nat. Prod. Rep.* **30**, 108–160 (2013).

43. Dong, S.-H. *et al.* The enterococcal cytolysin synthetase has an unanticipated lipid kinase fold. *Elife* **4**, (2015).

44. Ahmad, S. *et al.* The Natural Polypeptides as Significant Elastase Inhibitors. *Front. Pharmacol.* **11**, 688 (2020).

45. Freeman, M. F. *et al.* Metagenome mining reveals polytheonamides as posttranslationally modified ribosomal peptides. *Science* **338**, 387–390 (2012).

46. Bösch, N. M. *et al.* Landornamides: Antiviral Ornithine-Containing Ribosomal Peptides Discovered through Genome Mining. *Angew. Chem. Int. Ed Engl.* **59**, 11763–11768 (2020).

47. Motamedi, H. *et al.* Characterization of methyltransferase and hydroxylase genes involved in the biosynthesis of the immunosuppressants FK506 and FK520. *Journal of Bacteriology* vol. 178 5243–5248 (1996).

48. Labby, K. J., Watsula, S. G. & Garneau-Tsodikova, S. Interrupted adenylation domains: unique bifunctional enzymes involved in nonribosomal peptide biosynthesis. *Nat. Prod. Rep.* **32**, 641–653 (2015).

49. Song, H. & Naismith, J. H. Enzymatic methylation of the amide bond. *Curr. Opin. Struct. Biol.* **65**, 79–88 (2020).

50. van der Velden, N. S. *et al.* Autocatalytic backbone N-methylation in a family of ribosomal peptide natural products. *Nat. Chem. Biol.* **13**, 833–835 (2017).

51. Miller, F. S. *et al.* Conformational rearrangements enable iterative backbone N-methylation in RiPP biosynthesis. *Nat. Commun.* **12**, 1–14 (2021).

52. Chatterjee, J., Rechenmacher, F. & Kessler, H. N-methylation of peptides and proteins: an important element for modulating biological functions. *Angew. Chem. Int. Ed Engl.* **52**, 254–269 (2013).

53. Nurk, S., Meleshko, D., Korobeynikov, A. & Pevzner, P. A. metaSPAdes: a new versatile metagenomic assembler. *Genome Res.* **27**, 824–834 (2017).

54. Li, H. & Durbin, R. Fast and accurate short read alignment with Burrows-Wheeler transform. *Bioinformatics* **25**, 1754–1760 (2009).

55. Kang, D. D. *et al.* MetaBAT 2: an adaptive binning algorithm for robust and efficient genome reconstruction from metagenome assemblies. *PeerJ* **7**, e7359 (2019).

56. Meyer, F. *et al.* AMBER: Assessment of Metagenome BinnERs. *Gigascience* **7**, (2018).

57. Meyer, F. *et al.* Critical Assessment of Metagenome Interpretation - the second round of challenges. *bioRxiv* 2021.07.12.451567 (2021) doi:10.1101/2021.07.12.451567.

58. Parks, D. H., Imelfort, M., Skennerton, C. T., Hugenholtz, P. & Tyson, G. W. CheckM: assessing the quality of microbial genomes recovered from isolates, single cells, and metagenomes. *Genome Res.* **25**, 1043–1055 (2015).

59. Eren, A. M. *et al.* Anvi'o: an advanced analysis and visualization platform for 'omics data. *PeerJ* **3**, e1319 (2015).

60. Bowers, R. M. *et al.* Minimum information about a single amplified genome (MISAG) and a metagenome-assembled genome (MIMAG) of bacteria and archaea. *Nat. Biotechnol.* **35**, 725–731 (2017).

61. Olm, M. R., Brown, C. T., Brooks, B. & Banfield, J. F. dRep: a tool for fast and accurate genomic comparisons that enables improved genome recovery from metagenomes through de-replication. *ISME J.* **11**, 2864–2868 (2017).

62. Olm, M. R. *et al.* Consistent Metagenome-Derived Metrics Verify and Delineate Bacterial Species Boundaries. *mSystems* **5**, (2020).

63. Mende, D. R., Sunagawa, S., Zeller, G. & Bork, P. Accurate and universal delineation of prokaryotic species. *Nat. Methods* **10**, 881–884 (2013).

64. Chaumeil, P.-A., Mussig, A. J., Hugenholtz, P. & Parks, D. H. GTDB-Tk: a toolkit to classify genomes with the Genome Taxonomy Database. *Bioinformatics* (2019) doi:10.1093/bioinformatics/btz848.

65. Seemann, T. Prokka: rapid prokaryotic genome annotation. *Bioinformatics* **30**, 2068–2069 (2014).

66. Sunagawa, S. *et al.* Metagenomic species profiling using universal phylogenetic marker genes. *Nat. Methods* **10**, 1196–1199 (2013).

67. Huerta-Cepas, J. *et al.* Fast Genome-Wide Functional Annotation through Orthology Assignment by eggNOG-Mapper. *Mol. Biol. Evol.* **34**, 2115–2122 (2017).

68. Huerta-Cepas, J. *et al.* eggNOG 5.0: a hierarchical, functionally and phylogenetically annotated orthology resource based on 5090 organisms and 2502 viruses. *Nucleic Acids Res.* **47**, D309–D314 (2019).

69. Kanehisa, M. & Goto, S. KEGG: Kyoto Encyclopedia of Genes and Genomes. *Nucleic Acids Res.* **28**, 27–30 (2000).

70. Buchfink, B., Xie, C. & Huson, D. H. Fast and sensitive protein alignment using DIAMOND. *Nat. Methods* **12**, 59–60 (2015).

71. Tatusova, T. *et al.* NCBI prokaryotic genome annotation pipeline. *Nucleic Acids Res.* **44**, 6614–6624 (2016).

72. Blin, K. *et al.* antiSMASH 5.0: updates to the secondary metabolite genome mining pipeline. *Nucleic Acids Res.* **47**, W81–W87 (2019).

73. Fu, L., Niu, B., Zhu, Z., Wu, S. & Li, W. CD-HIT: accelerated for clustering the next-generation sequencing data. *Bioinformatics* **28**, 3150–3152 (2012).

74. Milanese, A. *et al.* Microbial abundance, activity and population genomic profiling with mOTUs2. *Nat. Commun.* **10**, 1014 (2019).

75. Kautsar, S. A., van der Hooft, J. J. J., de Ridder, D. & Medema, M. H. BiG-SLiCE: A highly scalable tool maps the diversity of 1.2 million biosynthetic gene clusters. *Gigascience* **10**, (2021).

76. Navarro-Muñoz, J. C. *et al.* A computational framework to explore large-scale biosynthetic diversity. *Nat. Chem. Biol.* **16**, 60–68 (2020).

77. Becht, E. *et al.* Dimensionality reduction for visualizing single-cell data using UMAP. *Nat. Biotechnol.* (2018) doi:10.1038/nbt.4314.

78. McInnes, L., Healy, J. & Astels, S. hdbscan: Hierarchical density based clustering.

Journal of Open Source Software **2**, 205 (2017).

79. Barco, R. A. *et al.* A Genus Definition for and Based on a Standard Genome Relatedness Index. *MBio* **11**, (2020).

80. Nguyen, L.-T., Schmidt, H. A., von Haeseler, A. & Minh, B. Q. IQ-TREE: a fast and effective stochastic algorithm for estimating maximum-likelihood phylogenies. *Mol. Biol. Evol.* **32**, 268–274 (2015).

81. Edgar, R. C. MUSCLE: multiple sequence alignment with high accuracy and high throughput. *Nucleic Acids Res.* **32**, 1792–1797 (2004).

82. Capella-Gutiérrez, S., Silla-Martínez, J. M. & Gabaldón, T. trimAl: a tool for automated alignment trimming in large-scale phylogenetic analyses. *Bioinformatics* **25**, 1972–1973 (2009).

83. Weimann, A. *et al.* From Genomes to Phenotypes: Traitar, the Microbial Trait Analyzer. *mSystems* **1**, (2016).

84. Pasternak, Z. *et al.* By their genes ye shall know them: genomic signatures of predatory bacteria. *ISME J.* **7**, 756–769 (2013).

85. Chen, F., Mackey, A. J., Stoeckert, C. J., Jr & Roos, D. S. OrthoMCL-DB: querying a comprehensive multi-species collection of ortholog groups. *Nucleic Acids Res.* **34**, D363–8 (2006).

86. Abby, S. S. & Rocha, E. P. C. Identification of Protein Secretion Systems in Bacterial Genomes Using MacSyFinder. *Methods Mol. Biol.* **1615**, 1–21 (2017).

87. Carradec, Q. *et al.* A global ocean atlas of eukaryotic genes. *Nat. Commun.* **9**, 373 (2018).

88. Liao, Y., Smyth, G. K. & Shi, W. featureCounts: an efficient general purpose program for assigning sequence reads to genomic features. *Bioinformatics* **30**, 923–930 (2014).

89. Bhushan, A., Egli, P. J., Peters, E. E., Freeman, M. F. & Piel, J. Genome mining- and synthetic biology-enabled production of hypermodified peptides. *Nat. Chem.* **11**, 931–939 (2019).

90. Bobeica, S. C. *et al.* Insights into AMS/PCAT transporters from biochemical and structural characterization of a double Glycine motif protease. *Elife* **8**, (2019).

91. Bode, E. *et al.* Promoter Activation in Δhfq Mutants as an Efficient Tool for Specialized Metabolite Production Enabling Direct Bioactivity Testing. *Angew. Chem. Int. Ed Engl.* **58**, 18957–18963 (2019).

92. Morinaka, B. I. *et al.* RadicalS-Adenosyl Methionine Epimerases: Regioselective Introduction of DiverseD-Amino Acid Patterns into Peptide Natural Products. *Angewandte Chemie International Edition* vol. 53 8503–8507 (2014).

93. Morinaka, B. I. *et al.* Natural noncanonical protein splicing yields products with diverse β-amino acid residues. *Science* vol. 359 779–782 (2018).

94. Le Roux, F., Binesse, J., Saulnier, D. & Mazel, D. Construction of a Vibrio splendidus mutant lacking the metalloprotease gene vsm by use of a novel counterselectable suicide vector. *Appl. Environ. Microbiol.* **73**, 777–784 (2007).

95. Thoma, S. & Schobert, M. An improved Escherichia coli donor strain for diparental mating. *FEMS Microbiol. Lett.* **294**, 127–132 (2009).

96. Minh, B. Q. *et al.* IQ-TREE 2: New Models and Efficient Methods for Phylogenetic Inference in the Genomic Era. *Mol. Biol. Evol.* **37**, 1530–1534 (2020).

97. Hoang, D. T., Chernomor, O., von Haeseler, A., Minh, B. Q. & Vinh, L. S. UFBoot2: Improving the Ultrafast Bootstrap Approximation. *Mol. Biol. Evol.* **35**, 518–522 (2018).

98. Conway, J. R., Lex, A. & Gehlenborg, N. UpSetR: an R package for the visualization of intersecting sets and their properties. *Bioinformatics* **33**, 2938–2940 (2017).

99. Yu, G., Smith, D. K., Zhu, H., Guan, Y. & Lam, T. T.-Y. Ggtree : An r package for visualization and annotation of phylogenetic trees with their covariates and other associated data. *Methods Ecol. Evol.* **8**, 28–36 (2017).

100. Tully, B. J., Graham, E. D. & Heidelberg, J. F. The reconstruction of 2,631 draft metagenome-assembled genomes from the global oceans. *Sci Data* **5**, 170203 (2018).

101. Steinegger, M. & Söding, J. MMseqs2 enables sensitive protein sequence searching for the analysis of massive data sets. *Nat. Biotechnol.* **35**, 1026–1028 (2017).

102. Katoh, K. & Standley, D. M. MAFFT Multiple Sequence Alignment Software Version 7: Improvements in Performance and Usability. *Molecular Biology and Evolution* vol. 30 772–780 (2013).

103. Price, M. N., Dehal, P. S. & Arkin, A. P. FastTree 2--approximately maximum-likelihood trees for large alignments. *PLoS One* **5**, e9490 (2010).

104. Conibear, A. C., Rosengren, K. J., Becker, C. F. W. & Kaehlig, H. Random coil shifts of posttranslationally modified amino acids. *J. Biomol. NMR* **73**, 587–599 (2019).

105. Gallis, D. E. & Crist, D. R. Use of NOE difference spectra to determine configurations and conformations of imidate esters. *Magnetic Resonance in Chemistry* vol. 25 480–483 (1987).

106. Meese, C. O. & Walter, W. Unusual13C/77se couplings in the13C NMR spectra of selenoimidates. *Magnetic Resonance in Chemistry* vol. 23 327–329 (1985).

107. Meese, C. O., Specht, D. & Hofmann, U. Syntheses of metabolites of S-carboxymethyl-L-cysteine and S-methyl-L-cysteine and of some isotopically labelled (2H, 13C) analogues. *Arch. Pharm.* **323**, 957–965 (1990).

108. Mordhorst, S., Siegrist, J., Müller, M., Richter, M. & Andexer, J. N. Catalytic Alkylation Using a Cyclic S-Adenosylmethionine Regeneration System. *Angew. Chem. Int. Ed Engl.* **56**, 4037–4041 (2017).

109. Sterner, O., Etzel, W., Mayer, A. & Anke, H. Omphalotin, A New Cyclic Peptide with Potent Nematicidal Activity fromOmphalotus OleariusII. Isolation and Structure Determination. *Natural Product Letters* vol. 10 33–38 (1997).

110. Maguire, F. *et al.* Metagenome-assembled genome binning methods with short reads disproportionately fail for plasmids and genomic Islands. *Microb Genom* **6**, (2020).

111. Carattoli, A. *et al.* In silico detection and typing of plasmids using PlasmidFinder and plasmid multilocus sequence typing. *Antimicrob. Agents Chemother.* **58**, 3895–3903 (2014).

112. Krawczyk, P. S., Lipinski, L. & Dziembowski, A. PlasFlow: predicting plasmid sequences in metagenomic data using genome signatures. *Nucleic Acids Res.* **46**, e35 (2018).

113. Zhou, F. & Xu, Y. cBar: a computer program to distinguish plasmid-derived from chromosome-derived sequence fragments in metagenomics data. *Bioinformatics* **26**, 2051–2052 (2010).

114. Roux, S., Enault, F., Hurwitz, B. L. & Sullivan, M. B. VirSorter: mining viral signal from microbial genomic data. *PeerJ* **3**, e985 (2015).

115. Ren, J. *et al.* Identifying viruses from metagenomic data using deep learning. *Quantitative Biology* vol. 8 64–77 (2020).

116. West, P. T., Probst, A. J., Grigoriev, I. V., Thomas, B. C. & Banfield, J. F. Genome-reconstruction for eukaryotes from complex natural microbial communities. *Genome Res.* **28**, 569–580 (2018).

117. Shaiber, A. & Eren, A. M. Composite Metagenome-Assembled Genomes Reduce the Quality of Public Genome Repositories. *mBio* vol. 10 (2019).

118. Chen, L.-X., Anantharaman, K., Shaiber, A., Eren, A. M. & Banfield, J. F. Accurate and complete genomes from metagenomes. *Genome Res.* **30**, 315–333 (2020).

119. Mende, D. R. *et al.* proGenomes2: an improved database for accurate and consistent habitat, taxonomic and functional annotations of prokaryotic genomes. *Nucleic Acids Res.* **48**, D621–D625 (2020).

120. Carroll, L. M. *et al.* Accurate de novo identification of biosynthetic gene clusters with GECCO. *bioRxiv* 2021.05.03.442509 (2021) doi:10.1101/2021.05.03.442509.

121. Lee, Y.-J. *et al.* Identification and biosynthesis of thymidine hypermodifications in the genomic DNA of widespread bacterial viruses. *Proc. Natl. Acad. Sci. U. S. A.* **115**, E3116–E3125 (2018).

122. Weigele, P. & Raleigh, E. A. Biosynthesis and Function of Modified Bases in Bacteria and Their Viruses. *Chem. Rev.* **116**, 12655–12687 (2016).

123. Sutherland, K. M., Wankel, S. D. & Hansel, C. M. Dark biological superoxide production as a significant flux and sink of marine dissolved oxygen. *Proc. Natl. Acad. Sci. U. S. A.* **117**, 3433–3439 (2020).

124. Pastor, J. M. *et al.* Ectoines in cell stress protection: uses and biotechnological production. *Biotechnol. Adv.* **28**, 782–801 (2010).

125. Moore, C. M. *et al.* Processes and patterns of oceanic nutrient limitation. *Nat. Geosci.* **6**, 701–710 (2013).

126. Allemann, M. N., Shulse, C. N. & Allen, E. E. Linkage of Marine Bacterial Polyunsaturated Fatty Acid and Long-Chain Hydrocarbon Biosynthesis. *Front. Microbiol.* **10**, 702 (2019).

127. Nakai, R. Size Matters: Ultra-small and Filterable Microorganisms in the Environment. *Microbes Environ.* **35**, (2020).

128. Lang, A. S., Zhaxybayeva, O. & Beatty, J. T. Gene transfer agents: phage-like elements of genetic exchange. *Nat. Rev. Microbiol.* **10**, 472–482 (2012).

129. Lannes, R., Olsson-Francis, K., Lopez, P. & Bapteste, E. Carbon Fixation by Marine Ultrasmall Prokaryotes. *Genome Biol. Evol.* **11**, 1166–1177 (2019).

130. Castelle, C. J. *et al.* Biosynthetic capacity, metabolic variety and unusual biology in the CPR and DPANN radiations. *Nat. Rev. Microbiol.* **16**, 629–645 (2018).

131. Karasikov, M. *et al.* MetaGraph: Indexing and Analysing Nucleotide Archives at Petabase-scale. *bioRxiv* 2020.10.01.322164 (2020) doi:10.1101/2020.10.01.322164.

132. Murray, A. E. *et al.* Roadmap for naming uncultivated Archaea and Bacteria. *Nat Microbiol* **5**, 987–994 (2020).

133. Xiao, Y., Wei, X., Ebright, R. & Wall, D. Antibiotic Production by Myxobacteria Plays a Role in Predation. *Journal of Bacteriology* vol. 193 4626–4633 (2011).

134. Widderich, N. *et al.* Biochemical properties of ectoine hydroxylases from extremophiles and their wider taxonomic distribution among microorganisms. *PLoS One* **9**, e93809 (2014).

135. Funa, N., Ozawa, H., Hirata, A. & Horinouchi, S. Phenolic lipid synthesis by type III polyketide synthases is essential for cyst formation in Azotobacter vinelandii. *Proc. Natl. Acad. Sci. U. S. A.* **103**, 6356–6361 (2006).

136. Funabashi, M. *et al.* The biosynthesis of liposidomycin-like A-90289 antibiotics featuring a new type of sulfotransferase. *Chembiochem* **11**, 184–190 (2010).

137. Grubbs, K. J. *et al.* Large-Scale Bioinformatics Analysis of Bacillus Genomes

Uncovers Conserved Roles of Natural Products in Bacterial Physiology. *mSystems* **2**, (2017).

138. Allen, E. E. & Bartlett, D. H. Structure and regulation of the omega-3 polyunsaturated fatty acid synthase genes from the deep-sea bacterium Photobacterium profundum strain SS9The GenBank accession numbers for the sequences reported in this paper are AF409100 and AF467805. *Microbiology* **148**, 1903–1913 (2002).

139. Sukovich, D. J., Seffernick, J. L., Richman, J. E., Gralnick, J. A. & Wackett, L. P. Widespread Head-to-Head Hydrocarbon Biosynthesis in Bacteria and Role of OleA. *Appl. Environ. Microbiol.* **76**, 3850–3862 (2010).

140. Montalbán-López, M. *et al.* New developments in RiPP discovery, enzymology and engineering. *Nat. Prod. Rep.* (2020) doi:10.1039/d0np00027b.

141. Mullowney, M. W., McClure, R. A., Robey, M. T., Kelleher, N. L. & Thomson, R. J. Natural products from thioester reductase containing biosynthetic pathways. *Nat. Prod. Rep.* **35**, 847–878 (2018).

142. Prieto, C., García-Estrada, C., Lorenzana, D. & Martín, J. F. NRPSsp: non-ribosomal peptide synthase substrate predictor. *Bioinformatics* **28**, 426–427 (2011).

143. Parker, J. B. & Walsh, C. T. Action and timing of BacC and BacD in the late stages of biosynthesis of the dipeptide antibiotic bacilysin. *Biochemistry* **52**, 889–901 (2013).

144. Hamada, T., Matsunaga, S., Yano, G. & Fusetani, N. Polytheonamides A and B, highly cytotoxic, linear polypeptides with unprecedented structural features, from the marine sponge, Theonella swinhoei. *J. Am. Chem. Soc.* **127**, 110–118 (2005).

145. Johnson, M. *et al.* NCBI BLAST: a better web interface. *Nucleic Acids Res.* **36**, W5–9 (2008).

146. Merwin, N. J. *et al.* DeepRiPP integrates multiomics data to automate discovery of novel ribosomally synthesized natural products. *Proc. Natl. Acad. Sci. U. S. A.* **117**, 371–380 (2020).

147. Repka, L. M., Chekan, J. R., Nair, S. K. & van der Donk, W. A. Mechanistic Understanding of Lanthipeptide Biosynthetic Enzymes. *Chem. Rev.* **117**, 5457–5520 (2017).

148. Kelley, L. A., Mezulis, S., Yates, C. M., Wass, M. N. & Sternberg, M. J. E. The Phyre2 web portal for protein modeling, prediction and analysis. *Nat. Protoc.* **10**, 845–858 (2015).

149. Freeman, M. F., Helf, M. J., Bhushan, A., Morinaka, B. I. & Piel, J. Seven enzymes create extraordinary molecular complexity in an uncultivated bacterium. *Nature Chemistry* vol. 9 387–395 (2017).

150. Ma, H. *et al.* Dissecting the catalytic and substrate binding activity of a class II lanthipeptide synthetase BovM. *Biochem. Biophys. Res. Commun.* **450**, 1126–1132 (2014).

151. You, Y. O. & van der Donk, W. A. Mechanistic investigations of the dehydration reaction of lacticin 481 synthetase using site-directed mutagenesis. *Biochemistry* **46**, 5991–6000 (2007).

152. Kloosterman, A. M., Shelton, K. E., van Wezel, G. P., Medema, M. H. & Mitchell, D. A. RRE-Finder: a Genome-Mining Tool for Class-Independent RiPP Discovery. *mSystems* **5**, (2020).

153. Burkhart, B. J., Hudson, G. A., Dunbar, K. L. & Mitchell, D. A. A prevalent peptide-binding domain guides ribosomal natural product biosynthesis. *Nat. Chem. Biol.* **11**, 564–570 (2015).

154. Kandathil, S. M., Greener, J. G. & Jones, D. T. Recent developments in deep learning applied to protein structure prediction. *Proteins* **87**, 1179–1189 (2019).

155. Krogh, A., Larsson, B., von Heijne, G. & Sonnhammer, E. L. Predicting transmembrane protein topology with a hidden Markov model: application to complete genomes. *J. Mol. Biol.* **305**, 567–580 (2001).

156. Aramini, J. M. *et al.* Solution NMR structure of the NlpC/P60 domain of lipoprotein Spr from Escherichia coli: structural evidence for a novel cysteine peptidase catalytic triad. *Biochemistry* **47**, 9715–9717 (2008).

157. Siodłak, D. α,β-Dehydroamino acids in naturally occurring peptides. *Amino Acids* **47**, 1–17 (2015).

158. Butler, E. *et al.* Synthesis of macrocyclic precursors of the vioprolides. *Org. Biomol. Chem.* **16**, 6935–6960 (2018).

159. Dugave, C. & Demange, L. Cis-trans isomerization of organic molecules and biomolecules: implications and applications. *Chem. Rev.* **103**, 2475–2532 (2003).

160. Jensen, M. R. & Freeman, M. F. Structure and Biosynthesis of Proteusin RiPP Natural Products. *Comprehensive Natural Products III* 88–118 (2020) doi:10.1016/b978-0-12-409547-2.14727-4.

161. Renevey, A. & Riniker, S. The importance of N-methylations for the stability of the $\beta^{6.3}$-helical conformation of polytheonamide B. *Eur. Biophys. J.* **46**, 363–374 (2017).

162. Siegrist, J. *et al.* Regiocomplementary O-Methylation of Catechols by Using Three-Enzyme Cascades. *Chembiochem* **16**, 2576–2579 (2015).

163. Shafiee, A., Motamedi, H. & Chen, T. Enzymology of FK-506 biosynthesis. Purification and characterization of 31-O-desmethylFK-506 O:methyltransferase from Streptomyces sp. MA6858. *Eur. J. Biochem.* **225**, 755–764 (1994).

164. Wiebach, V. *et al.* An Amphipathic Alpha-Helix Guides Maturation of the

Ribosomally-Synthesized Lipolanthines. *Angew. Chem. Int. Ed Engl.* **59**, 16777–16785 (2020).

165. Morinaka, B. I., Verest, M., Freeman, M. F., Gugger, M. & Piel, J. An Orthogonal D O-Based Induction System that Provides Insights into d-Amino Acid Pattern Formation by Radical S-Adenosylmethionine Peptide Epimerases. *Angew. Chem. Int. Ed Engl.* **56**, 762–766 (2017).

166. Lanz, N. D. *et al.* Enhanced Solubilization of Class B RadicalS-Adenosylmethionine Methylases by Improved Cobalamin Uptake inEscherichia coli. *Biochemistry* vol. 57 1475–1490 (2018).

167. Giel, J. L. *et al.* Regulation of iron-sulphur cluster homeostasis through transcriptional control of the Isc pathway by [2Fe-2S]-IscR inEscherichia coli. *Molecular Microbiology* vol. 87 478–492 (2013).
