# Supplementary material for: Biosynthetic potential of the global ocean microbiome
Source: Nature. 2022 Jun 22;607(7917):111–8. doi: 10.1038/s41586-022-04862-3 (PMC9259500; doi:10.1038/s41586-022-04862-3)
Supplement: Supplementary file 2 — Reporting Summary [file 41586_2022_4862_MOESM2_ESM.pdf]

nature portfolio

Corresponding author(s): Shinichi Sunagawa, Joern Piel, Serina L. Robinson

Last updated by author(s): Mar 28, 2021

Reporting Summary

Nature Portfolio wishes to improve the reproducibility of the work that we publish. This form provides structure for consistency and transparency in reporting. For further information on Nature Portfolio policies, see our Editorial Policies and the Editorial Policy Checklist.

Statistics

For all statistical analyses, confirm that the following items are present in the figure legend, table legend, main text, or Methods section.

n/a	Confirmed	
☐	☒	The exact sample size (n) for each experimental group/condition, given as a discrete number and unit of measurement
☐	☒	A statement on whether measurements were taken from distinct samples or whether the same sample was measured repeatedly
☐	☒	The statistical test(s) used AND whether they are one- or two-sided<br>*Only common tests should be described solely by name; describe more complex techniques in the Methods section.*
☒	☐	A description of all covariates tested
☐	☒	A description of any assumptions or corrections, such as tests of normality and adjustment for multiple comparisons
☐	☒	A full description of the statistical parameters including central tendency (e.g. means) or other basic estimates (e.g. regression coefficient) AND variation (e.g. standard deviation) or associated estimates of uncertainty (e.g. confidence intervals)
☐	☒	For null hypothesis testing, the test statistic (e.g. F, t, r) with confidence intervals, effect sizes, degrees of freedom and P value noted<br>*Give P values as exact values whenever suitable.*
☒	☐	For Bayesian analysis, information on the choice of priors and Markov chain Monte Carlo settings
☒	☐	For hierarchical and complex designs, identification of the appropriate level for tests and full reporting of outcomes
☐	☒	Estimates of effect sizes (e.g. Cohen's d, Pearson's r), indicating how they were calculated

Our web collection on statistics for biologists contains articles on many of the points above.

Software and code

Policy information about availability of computer code

Data collection	Data collected from ENA was downloaded from ENA using their API. All other publicly available data was downloaded directly from the sources specified in the data availability statement.
Data analysis	The data was analyzed using a pipeline developed as part of this study as well as with additional ad hoc python and R scripts and with the following softwares: BBMap v.38.71, metaSPAdes v3.11.1 and v3.12, BWA v0.7.17-r1188, MetaBAT2 v2.12.1, CheckM v1.0.13, Anvi'o v5.5.0, dRep v2.5.4, SpecI, GTDB-Tk v1.0.2, Prokka v1.14.5, fetchMGs v1.2, emapper v2.0.1, DIAMOND v0.9.30, antiSMASH v5.1.0 and v5.0.0, CD-HIT v4.8.1, mOTUs v2.5.1, BiG-SLICE v1.1, IQTREE v2.0.3, MUSCLE v3.8.1551, trimal v1.4.1, Traitar v1.1.2, TXSSCAN v1.0.2, FeatureCounts v2.0.1, PlsamidFinder v2.1, PlasFlow v1.1.0, cBar v1.2, VirSorter v1.0.5, DeepVirFinder v1.0, EukRep v0.6.6, ccontigs v1.0.0, STAG v0.7, GECCO v0.4.4, MMSEQS2 v13.45111, MAFFT v7.310, Python >= 3.6 with the packages pandas (v1.0.0-1.3.4), biopython (v1.73), umap-learn (v0.5.2), hdbscan (v0.8.28), scikit-learn (v1.0.2) and R (v4.0.0-v4.1.2) with the packages ggplot2 (v3.3.0-v3.3.5), tidyverse (v1.3.1), vegan (v2.5.7), ggtree (v3.3.0.901) and tidytree (v0.3.6), treeio (v1.19.1) and UpSetR (v1.4.0).<br>The code used in this study is accessible at https://github.com/SushiLab/magpipe/ and archived at Zenodo (https://doi.org/10.5281/zenodo.6393817).

For manuscripts utilizing custom algorithms or software that are central to the research but not yet described in published literature, software must be made available to editors and reviewers. We strongly encourage code deposition in a community repository (e.g. GitHub). See the Nature Portfolio guidelines for submitting code & software for further information.

Data

Policy information about availability of data

All manuscripts must include a data availability statement. This statement should provide the following information, where applicable:

- Accession codes, unique identifiers, or web links for publicly available datasets
- A description of any restrictions on data availability
- For clinical datasets or third party data, please ensure that the statement adheres to our policy

The metagenomic and metatranscriptomic data used in this study was downloaded from the European Nucleotide Archive (ENA) and their accessions are summarized in Supplementary Table 1. Publicly available genomes were downloaded from https://doi.org/10.6084/m9.figshare.4902923 for manually curated MAGs from Tara Oceans, from ENA using the project accession PRJEB33281 for GORG and from https://mmp2.sfb.uit.no/databases/ for MarDB. The GEM MAGs were downloaded from https://portal.nersc.gov/GEM/. MAGs contained in the GTDB r89 were downloaded from https://data.gtdb.ecogenomic.org/releases/release89/. The MIBiG and BiG-FAM databases can be accessed at https://mibig.secondarymetabolites.org/ and https://bigfam.bioinformatics.nl/, respectively. The data produced in this study, including metagenomic assemblies, bins and MAGs have been deposited at the European Nucleotide Archive under the accession PRJEB45951 and individual accessions are summarized in Supplementary Table 1. Other supporting data has been deposited on Zenodo (https://doi.org/10.5281/zenodo.4474310), and the OMD can be interactively accessed at https://microbiomics.io/ocean/. Additional material generated in this study is available upon request.

Field-specific reporting

Please select the one below that is the best fit for your research. If you are not sure, read the appropriate sections before making your selection.

☒ Life sciences ☐ Behavioural & social sciences ☐ Ecological, evolutionary & environmental sciences

For a reference copy of the document with all sections, see nature.com/documents/nr-reporting-summary-flat.pdf

Life sciences study design

All studies must disclose on these points even when the disclosure is negative.

Sample size	Samples sizes were defined by the availability of published data that were used to perform the analyses.
Data exclusions	No data were excluded from the analyses.
Replication	All post-translational modification of the peptides reported in this study were supported by replicated experiments, bio-activity assays included replicates (n >= 3) and all replicates were successful.
Randomization	For the different analyses conducted in this study, all samples were processed similarly and thus randomization was not necessary.
Blinding	For the different analyses conducted in this study, all samples were processed similarly and thus blinding was not necessary.

Reporting for specific materials, systems and methods

We require information from authors about some types of materials, experimental systems and methods used in many studies. Here, indicate whether each material, system or method listed is relevant to your study. If you are not sure if a list item applies to your research, read the appropriate section before selecting a response.

Materials & experimental systems

n/a	Involved in the study	
☒	☐	Antibodies
☐	☒	Eukaryotic cell lines
☒	☐	Palaeontology and archaeology
☒	☐	Animals and other organisms
☒	☐	Human research participants
☒	☐	Clinical data
☒	☐	Dual use research of concern

Methods

n/a	Involved in the study	
☒	☐	ChIP-seq
☒	☐	Flow cytometry
☒	☐	MRI-based neuroimaging

Eukaryotic cell lines

Policy information about cell lines

Cell line source(s)	HeLa cells.

Authentication	Not authenticated.
Mycoplasma contamination	Not tested.
Commonly misidentified lines<br>(See ICLAC register)	Not applicable.
